# Construction of Bone Metastasis-Specific Regulation Network Based on Prognostic Stemness-Related Signatures in Breast Invasive Carcinoma

**DOI:** 10.3389/fonc.2020.613333

**Published:** 2021-01-27

**Authors:** Runzhi Huang, Zhenyu Li, Jiayao Zhang, Zhiwei Zeng, Jiaqi Zhang, Mingxiao Li, Siqao Wang, Shuyuan Xian, Yuna Xue, Xi Chen, Jie Li, Wenjun Cheng, Bin Wang, Penghui Yan, Daoke Yang, Zongqiang Huang

**Affiliations:** ^1^Department of Orthopedics, The First Affiliated Hospital of Zhengzhou University, Zhengzhou, China; ^2^Division of Spine, Department of Orthopedics, Tongji Hospital Affiliated to Tongji University School of Medicine, Shanghai, China; ^3^Tongji University School of Medicine, Tongji University, Shanghai, China; ^4^Tongji University School of Mathematical Sciences, Tongji University, Shanghai, China; ^5^Department of General Surgery, Changzheng Hospital, Second Military Medical University, Shanghai, China; ^6^Department of Radiotherpy, The First Affiliated Hospital of Zhengzhou University, Zhengzhou, China

**Keywords:** breast invasive carcinoma, bone metastasis, apical junction, MAF, CD248, mRNA stemness index (mRNAsi), weighted gene co-expression network analysis (WGCNA), spatial transcriptome

## Abstract

**Background:**

Bone is the most common metastatic site of Breast invasive carcinoma (BRCA). In this study, the bone metastasis-specific regulation network of BRCA was constructed based on prognostic stemness-related signatures (PSRSs), their upstream transcription factors (TFs) and downstream pathways.

**Methods:**

Clinical information and RNA-seq data of 1,080 primary BRCA samples (1,048 samples without bone metastasis and 32 samples with bone metastasis) were downloaded from The Cancer Genome Atlas (TCGA). The edgeR method was performed to identify differential expressed genes (DEGs). Next, mRNA stemness index (mRNAsi) was calculated by one-class logistic regression (OCLR). To analyze DEGs by classification, similar genes were integrated into the same module by weighted gene co-expression network analysis (WGCNA). Then, univariate and multivariate Cox proportional hazard regression were applied to find the PSRSs. Furthermore, PSRSs, 318 TFs obtained from Cistrome database and 50 hallmark pathways quantified by GSVA were integrated into co-expression analysis. Significant co-expression patterns were used to construct the bone metastasis-specific regulation network. Finally, spatial single-cell RNA-seq and chromatin immunoprecipitation sequence (ChIP-seq) data and multi-omics databases were applied to validate the key scientific hypothesis in the regulation network. Additionally, Connectivity Map (CMap) was utilized to select the potential inhibitors of bone metastasis-specific regulation network in BRCA.

**Results:**

Based on edgeR and WGCNA method, 43 PSRSs were identified. In the bone metastasis-specific regulation network, MAF positively regulated CD248 (R = 0.435, P < 0.001), and hallmark apical junction was the potential pathway of CD248 (R = 0.353, P < 0.001). This regulatory pattern was supported by spatial single-cell RNA sequence, ChIP-seq data and multi-omics online databases. Additionally, alexidine was identified as the possible inhibitor for bone metastasis of BRCA by CMap analysis.

**Conclusion:**

PSRSs played important roles in bone metastasis of BRCA, and the prognostic model based on PSRSs showed good performance. Especially, we proposed that CD248 was the most significant PSRS, which was positively regulated by MAF, influenced bone metastasis *via* apical junction pathway. And this axis might be inhibited by alexidine, which providing a potential treatment strategy for bone metastasis of BRCA.

## Introduction

Breast Invasive Carcinoma (BRCA) was the most common tumor in female, which originated from ducts and acinar epithelium at all levels of the breast, and most patients suffered from malignant epithelial tumor. And the BRCA can be classified into several types according to the state of progesterone receptor (PR), estrogen receptor (ER), and ERBB2 receptor (HER2) in histological stratification, which was applied in clinical practice (1). Estimated by American Cancer Society (ACS), there were 279,100 new cases, and 42,690 new death BRCA patients in 2020 (2). Besides, the five-year survival rate for BRCA in stage I, II, III, and IV were 98, 92, 75, and 27% according to the statistic from 2009 to 2015, respectively (3). Although the five-year survival rate of primary BRCA was high, the five-year survival rate of bone metastasis was only 20%, and patients were trapped in a vicious cycle between osteolytic degeneration and proliferation of cancer cells (4). Besides, the osteolytic lesions like pain in bone, fractures, spinal compression and hypercalcemia leaded to poor survival quality and death (4).

Machine learning based on high-throughput data played an important role in prognosis of cancer, and new characters defined by algorithm like mRNA stemness index (mRNAsi) and stemness-related gene provided a new way for analysis (5). Recently, analysis on triple-negative breast cancer were launched to explore the key gene related to the stemness and find the target for further therapy (6). Therefore, molecules participated in the bone metastasis required to be explored, and corresponded biomarkers and possible mechanism needed to be drug for clinical strategy and therapy.

In this study, data of RNA-seq in BRCA were identified by edgeR, and differential expressed genes (DEGs) were performed by machine-learning algorithm to define the mRNAsi. Then, the profiling of DEGs were integrated into weighted gene co-expression network analysis (WGCNA) to classify similar genes into multiply modules and outline the phenotypic characteristics of modules. Besides, the mRNAsi and Hallmark gene sets were qualified to annotate modules. Then, the key module and genes most associated with mRNAsi were selected. Univariate and multivariate Cox regression analysis were applied to access the prognostic value of genes. What is more, based on the Pearson analysis for TF, genes, and Hallmark gene sets, a bone metastasis-specific network was constructed. The scientific hypothesis was determined by the correlation coefficient. Moreover, the CMap analysis was applied to find potential inhibitors for signal axis. Finally, spatial single-cell RNA sequence and chromatin immunoprecipitation sequence (ChIP-seq) data and multi-omics online databases were applied to validate the key scientific hypothesis in the regulation network. The bone metastasis-specific regulation network and inhibitors provided potential treatment strategy for bone metastasis of BRCA.

## Methods

### Data Acquisition

Based on the Cancer Genome Atlas (TCGA) database (https://portal.gdc.cancer.gov/), RNA-seq data of 1,080 primary BRCA samples were downloaded, including 1,048 samples without bone metastasis and 32 samples with bone metastasis. And the bone metastasis was diagnosed by imaging examinations like CT or PET-CT. Besides, demographics like age and gender, tumor information like TNM stage and grade, and follow-up data of all patients were also obtained from the TCGA database. Besides, samples without follow-up information were excluded.

### Differentially Expressed Genes Identification

The edgeR package was used to screen the RNA-seq data to define DEGs between primary BRCA patients with and without bone metastasis, and the criteria must fit following two points at the same time: the absolute value of log Fold Change (log FC) must more than 1, and the False Discovery Rate (FDR) must less than 0.05. Then, Gene Oncology (GO) and Kyoto Encyclopedia of Genes and Genomes (KEGG) enrichment analysis were utilized to annotate DEGs.

### mRNAsi

The mRNAsi was calculated by the one-class logistic regression (OCLR) machine-learning algorithm (7) based on the RNA-seq data of 1,080 primary BRCA samples.

### Weighted Gene Co-Expression Network Analysis

With the aim to holistically analyze DEGs, modules were classified by WGCNA (8) R package (http://www.r-project.org/), every single module gathered highly similar DEGs. Then based on RNA-seq profiling, the Pearson correlation analysis was utilized to construct the gene co-expression network. Additionally, the power function was applied to build a weighted adjacency matrix:

$$a_{ij}=\;{\left|s_{ij}\right|}^{\beta }$$

S_ij_ represented the Pearson correlation between gene i and j, a_ij_ represented the weighted network adjacency between gene i and j. And β equaled to 4 was the soft-threshold parameter set by pickSoft Threshold from WGCNA R package. What’s more, the application of soft-threshold parameter of weighted network make it possible to show the continuous variety of co-expression information in [0,1], and it might promote the idea of scale-free co-expression network come true. In addition, the correlation coefficients were utilized to construct hierarchical clustering, and a topological overlap method was performed to classify DEGs with the similar expression patterns into same module. Besides, the capacity of module must more than 20 genes, and when a certain module with less than 20 genes, similar modules will be merged.

Based on the H Collection of Molecular Signatures Database (MSigDB) v7.0 (https://www.gsea-msigdb.org/gsea/msigdb/genesets.jsp?collection=H) (9), 50 Hallmark gene sets were qualitied by the computational approach named Gene Set Variation Analysis (GSVA). And these Hallmark gene sets were related to biological process and states. Then, with the aim to annotate the specific phenotypic traits for module, 50 Hallmark gene sets and mRNAsi were defined as phenotypes for a co-analysis with modules. Besides, gene significance (GS) coefficients and *p* values were illustrated the correlation between DEGs and phenotypes. Similarly, module significance (MS) coefficients and *p* values showed the correlation between modules and phenotypes, and the MS was calculated from the average absolute GS for all genes in every single module. Moreover, the first principle component of module genes showed the gene expression level in the certain module, and module eigengenes (MEs) represented the first principle component. And module membership (MM) represented the correlation between gene to MEs. Next, mRNAsi was the key phenotype to choose the stemness-related module, and the largest MS with *p* value less than 0.05 were applied to determine the key module for next exploration. What’s more, the Hallmark gene set significantly related to the key module and *p* value less than 0.05 was selected as the key Hallmark gene set, and the key Hallmark gene set was defined as the key pathway for stemness-related signatures (SRSs). Besides, SRSs with MM >0.300 and GS >0.300 were obtained from the key module for further analysis.

### Multivariate Prognosis Model Construction

The univariate and multivariate Cox proportional hazard regression were applied to find prognostic stemness-related signatures (PSRSs). SRSs with *p* value <0.001 in univariate Cox proportional hazard regression were defined as PSRSs. Then, the LASSO regression analysis was applied to avoid the over-fitness. Then, residual PSRSs were integrated into the multivariate Cox regression model, and risk score for each BRCA patient was calculated by the formula:

$$Risk\;score_{m}=\beta_{1}\times gene_{1}+\beta_{2}\times gene_{2}+\;\beta_{3}\times gene_{3}\ldots \ldots +\beta_{n}\times gene_{n}$$

In the formula, “m” represented the number of each patient, “n” represented prognostic PSRS, and “β” represented the coefficient of each prognostic PSRS. Then, patients with BRCA were divided into low- and high-risk groups according to risk score. And the efficiency of risk score of the model was detected by Kaplan-Meier survival analysis. What’s more, and accuracy was detected by the Receiver Operating Characteristic Curve (ROC) curve and C-index. Finally, the demographic information, TNM stage and risk score were applied for correction, and univariate and multivariate Cox regression were performed to validate the independent prognostic value.

### Potential Signal Axis Identification

Based on the Cistrome database (http://cistrome.org/) (10), the list of 318 Translate factors (TFs) were downloaded. And edgeR was utilized to find differential expressed TFs. Then co-expression analysis for TFs and PSRSs by Pearson correlation analysis, and the significant paired TF-PSRSs were selected.

Aim to identify the significantly co-expressed pathway, the absolute quantification of 50 Hallmark gene sets between non-metastasis and metastasis patients was screened by GSVA. And to explore the up- and down-regulated pathways between non-metastasis and metastasis patients, Gene Set Enrichment Analysis (GSEA) was conducted based on the 50 gene sets of Hallmark (11). In addition, the intersection of GSVA, GSEA, and module phenotypic traits was defined as the key pathway. Then, the Pearson correlation analysis was utilized to analysis the interaction between Hallmark gene sets and PSRSs.

Eventually, the network based on TFs, PSRSs and Hallmark gene sets was constructed. And String database (12) was applied to plot a protein-protein interaction (PPI) network. Besides, the criteria for TF-PSRS paired was the absolute value of the correlation coefficient more than 0.400 and *p* value less than 0.05, for PSRS-Hallmark gene set was the absolute value of correlation coefficient more than 0.300 and *p* value less than 0.05.

### Connectivity Map Analysis

To expend the application of potential signal axis, inhibitors of the signal pathway were selected by the Connectivity Map (build 02) (CMap) (https://portals.broadinstitute.org/cmap/) (13). Then, inhibitors for BCRA were identified. Besides, the information like chemical structural formula and biologic function of inhibitor compounds were available from the mechanism of actions (MoA) (http://clue.io/) (14). Ultimately, the key inhibitor was found according to the TF, PSRS, and Hallmark gene set.

### Assay for Targeting Accessible-Chromatin With High-Throughout Sequencing-Seq and Chromatin Immunoprecipitation Sequence Validation

Assay for Targeting Accessible-Chromatin with high-throughout sequencing (ATAC-seq) and ChIP-seq data were used to validated the regulation mechanism of the network. ATAC-seq and ChIP-seq data were obtained from TCGA GDC (https://gdc.cancer.gov/about-data/publications/ATACseq-AWG) and Cistrome database (http://cistrome.org/), respectively (10, 15). WashU Epigenome Browser and Gviz package were used to visualize the binding peaks (16, 17).

### Spatial Transcriptome Validation

The regulatory relationship between TF and PSRS, PSRS and Hallmark gene set required to be validated the direct mechanism in the molecular experiment. When linked with single-cell RNA sequence data, spatial transcriptome data can validate the cell subtype localization of the key genes (https://support.10xgenomics.com/spatial-gene-expression/datasets/1.1.0/V1_Breast_Cancer_Block_A_Section_1). For quality control, only fit the following standards at the same time can be selected: genes must express in more than 3 single cells, gene counts more than 1, cell transcripts range from 1,500 to 100,000.

To integrate data analysis, the Seurat method was performed (18). Then, the “sctransform” algorithm was utilized for normalization. In addition, to identify variable genes and spatial-specific genes, “vst” and “markvariogram” method were utilized, respectively. Next, the principal component analysis (PCA) was performed based on variable genes (18). In addition, the jackstraw analysis was utilized to select the principal components (PCs), and *p* value must less than 0.05. Then, the further t-distributed Stochastic Neighbor Embedding (t-SNE), Haematoxylin and Eosin (HE) and UMAP (Uniform Manifold Approximation and Projection) were applied to identify the cell sub-cluster based on the PCs (resolution = 0.50) (19). And in sub-cluster, DEGs were filtered when the absolute value of log FC less than 0.5 and FDR more than 0.05. Moreover, the location and expression of DEGs were demonstrated in feature plots and violin plots, respectively. Besides, every cluster was annotated by scMatch (20), singleR (21), and CellMarker (22) databases. Aim to annotate single cells, 50 hallmark gene sets were performed to absolutely quantify the signaling pathway activity in each single cell.

### Multidimension Validation

To decrease the inherent defects of analysis in silicon, multiply online databases were utilized to validate the scientific hypothesis in several aspects. And top five genes in the key pathway selected by GeneCard (https://www.genecards.org/) were also validated with TFs and PSRSs.

Gene Expression Profilling Interactive Analysis (GEPIA) (23), Oncomine (24), PROGgeneV2 (25), UALCAN (26), Linkedomics (27), SurvExpress (28), cBioportal (29), Genotype-Tissue Expression (GTEx) (30) and UCSC xena (31) validated in gene level based, Cancer Cell Line Encyclopedia (CCLE) (32) validated in cancer cell line level, The human protein altas (33) validated in tissue level in BRCA patient. Finally, String database (12) was utilized to construct the Protein-Protein Interaction network.

### Statistics Analysis

The R software (www.r-project.org; version 3.6.1; Institute for Statistics and Mathematics, Vienna, Austria) was applied in all statistics analysis in our study, and two-sided *p* value <0.05 was determined as statistically significant.

## Results

### Differentially Expressed Genes Identification

**Figure 1:**
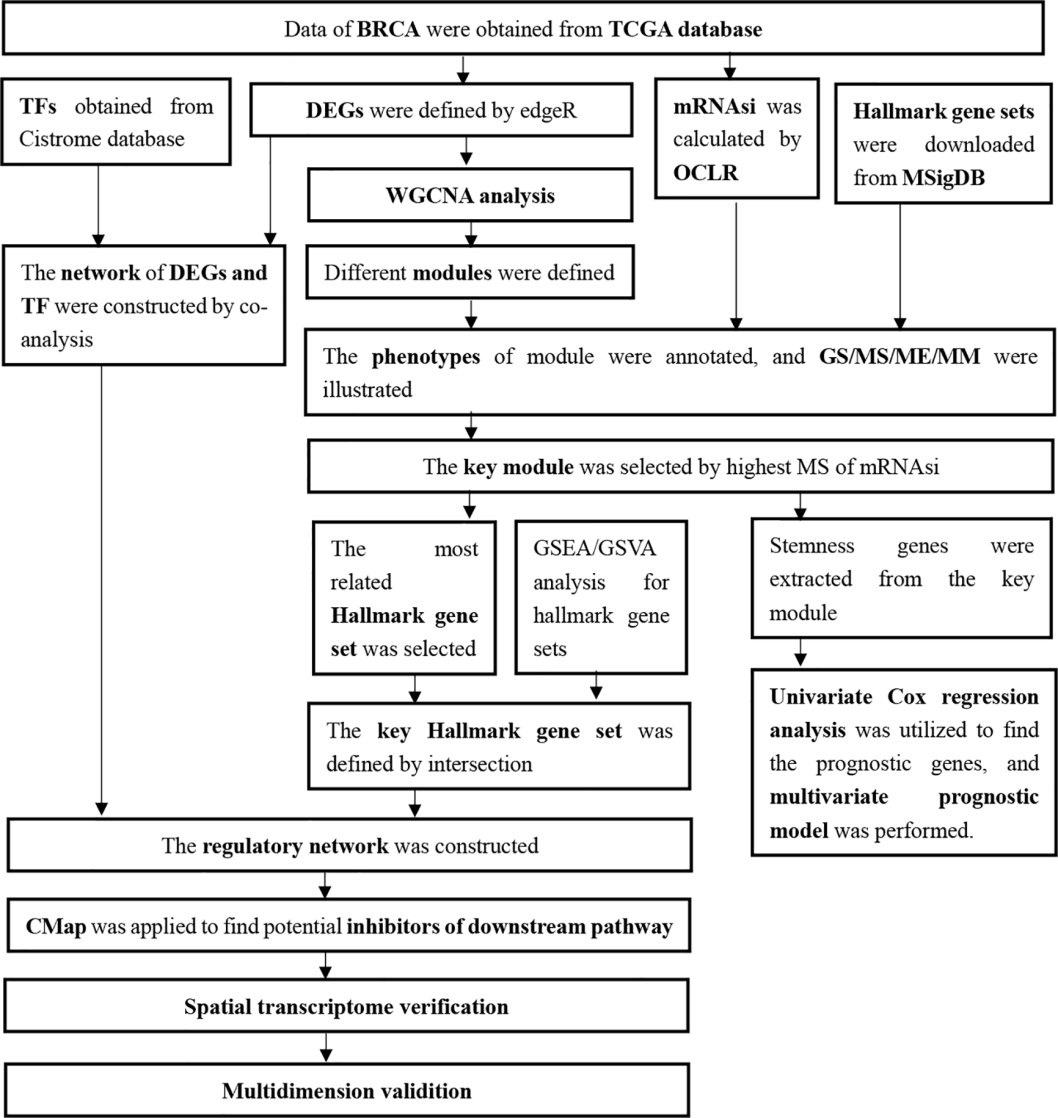
Flow chart of analysis.

The expression profiling of 1,048 primary BRCA samples without bone metastasis and 32 primary BRCA samples with bone metastasis were obtained from TCGA database, and all patients’ demographics information was summarized in **Table 1**. And all analysis processes were illustrated in **Figure 1**.

**Table 1:** Baseline information of 813 patients diagnosed with breast invasive carcinoma.

Variables	Total Patients (N = 813)
**Age, years**	
Mean ± SD	57.54 ± 12.66
Median (Range)	58 (26–90)
**T stage**	
T0	0
T1	222 (27.31%)
T2	477 (58.67%)
T3	88 (10.82%)
T4	26 (3.20%)
**N stage**	
N0	670 (82.41%)
N1	0
N2	92 (11.32%)
N3	51 (6.27%)
**M stage**	
M0	798 (98.15%)
M1	15 (1.85%)
**Stage**	
Stage I	152 (18.70%)
Stage II	468 (57.56%)
Stage III	178 (21.89%)
Stage IV	15 (1.85%)
**Metastasis**	
Bone metastasis	5 (0.62%)
Other metastasis	10 (1.23%)
No metastasis	798 (98.15%)

**Figure 2:**
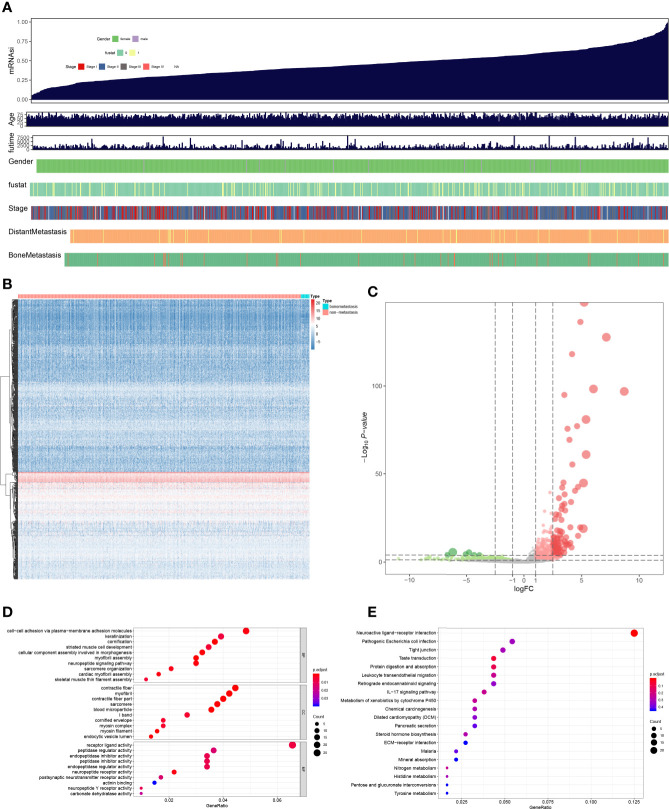
The summary of mRNAsi **(A)**, heatmap plot **(B)**, volcano plot **(C)**, GO **(D)**, and KEGG **(E)** enrichment analysis of differential expressed genes. Cell-cell adhesion *via* plasma-membrane adhesion molecules, contractile fiber, receptor ligand activity were the most significant GO items, and Neuroactive ligand-receptor interaction was the most significant KEGG item.

RNA-seq data from TCGA database was screened by edgeR to filter DEGs, and the information of BRCA (**Figure 2A**), the heatmap of RNA expression level in BRCA samples (**Figure 2B**) and volcano plots of DEGs and non-DEGs (**Figure 2C**) were launched. And 31 DEGs were founded. Besides, GO (**Figure 2D**) enrichment analysis for DEGs was performed, and cell-cell adhesion *via* plasma-membrane adhesion molecules (BP, GeneRatio = 0.048, *p* = 0.001, count = 21), contractile fiber (CC, GeneRatio = 0.045, *p* < 0.001, count = 20), receptor ligand activity (MF, GeneRatio = 0.066, *p* = 0.007, count = 27) were the most significant GO items. And KEGG (**Figure 2E**) enrichment analysis for DEGs showed Neuroactive ligand-receptor interaction (GeneRatio = 0.125, *p* = 0.001, count = 23) was the most significant KEGG item.

### WGCNA

**Figure 3:**
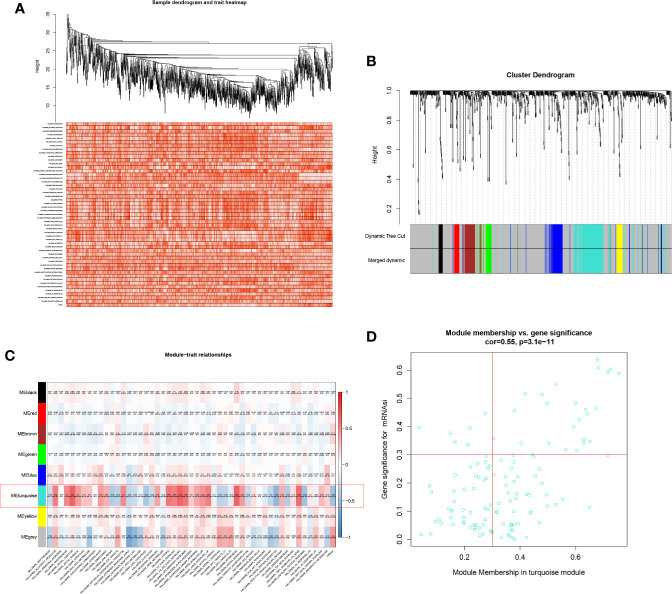
Heatmap of sample **(A)**, Cluster dendrogram of WGCNA **(B)**, co-expression heatmap of modules and phenotypes **(C)**, and the correlation between the mRNAsi and the module **(D)**. Module turquoise and hallmark apical junction was the key module and phenotype.

Based on WGGCNA package, seven modules were defined (**Figures 3A, B**). Aim to annotate the phenotype of modules, 50 Hallmark gene sets and mRNAsi were co-analysis with modules in the heatmap plot (**Figure 3C**). Module turquoise (MS = 0.550; *p* < 0.001) was the module most relevant to mRNAsi (MS = 0.670; *p* < 0.001) and 125 SRSs in turquoise were integrated into the further analysis. In addition, three Hallmark gene sets were highly correlated with module turquoise: hallmark apical junction (MS = 0.670; *p* < 0.001), hallmark myogenesis (MS = 0.66; *p* < 0.001), and hallmark IL6-JAK-STAT3 signaling (MS = 0.660; *p* < 0.001) (**Figure 3D**).

### Multivariate Prognostic Model Construction

**Figure 4:**
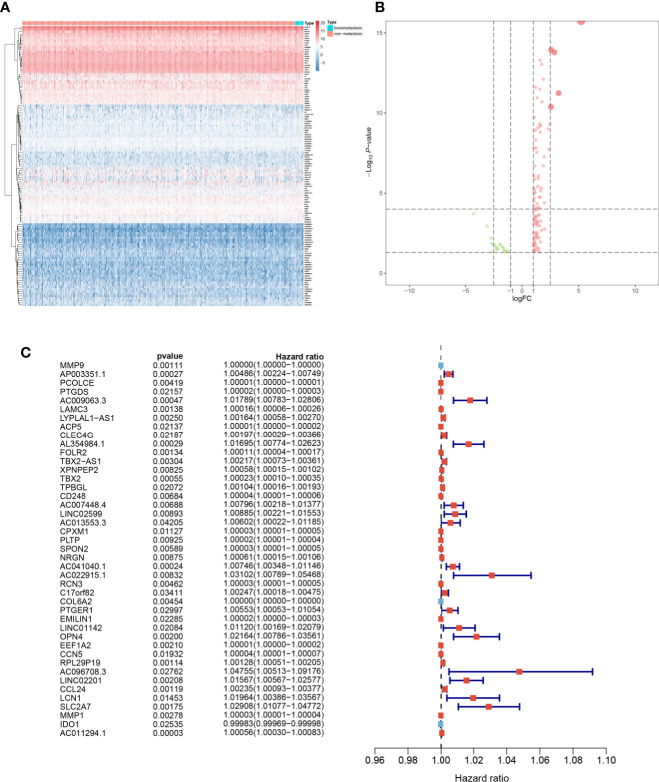
The heatmap **(A)**, volcano plot **(B)**, and forest plots **(C)** of key genes from key module.

**Figure 5:**
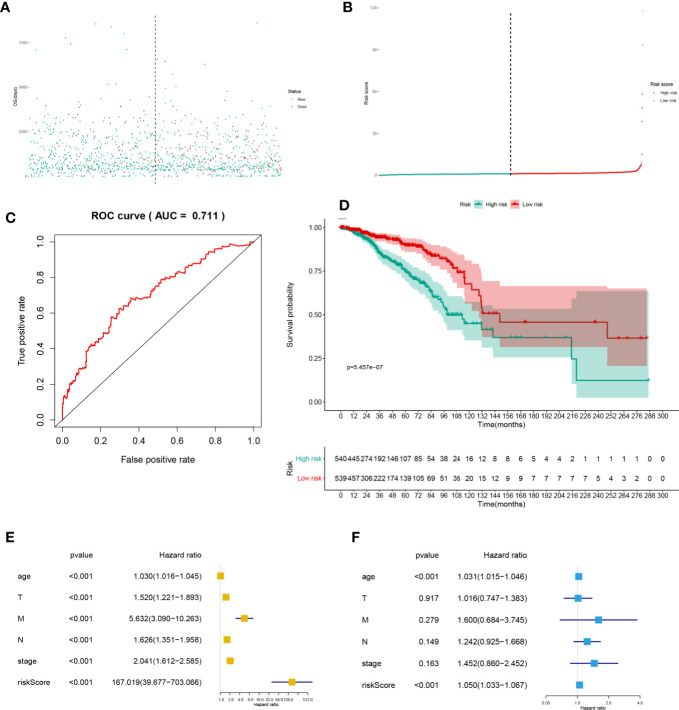
The scatter plot **(A)**, risk line plot **(B)**, ROC curve (AUC = 0.711) **(C)**, and Kaplan-Meier plot (*p* < 0.001) **(D)** for multivariate prognosis model. And univariate **(E)** and multivariate **(F)** Cox regression analysis for risk score. And the risk score was the independent predict factor.

The heatmap (**Figure 4A**) and volcano plot (**Figure 4B**) demonstrated the results of DEG analysis of 125 SRSs. And univariate Cox regression analysis was utilized to find the prognostic genes, and 43 PSRSs were identified. In addition, forest plot (**Figure 4C**) showed CD248 (HR = 1.00004, 95%CI (1.00001-1.00006), *p* = 0.007) was significantly associated with prognosis of BRCA. Then, the PSRSs were integrated into multivariate Cox regression analysis, and a prognostic model was constructed. The scatter plot (**Figure 5A**) and risk line plot (**Figure 5B**) were drawn to illustrated the risk distribution of each patient. In addition, in term of model diagnosis, area under curve (AUC) of ROC curve was 0.711 (**Figure 5C**). Based on the median risk score calculated by multivariate model, low- and high-risk groups were accessed by Kaplan-Meier survival analysis (**Figure 5D**), and the result displayed a significant difference (*p* < 0.001). Finally, the risk score was co-analysis with age, T stage, N stage, M stage, stage, the univariate (**Figure 5E**) (HR = 167.019, 95%CI (39.677–703.066), *p* < 0.001) and multivariate (**Figure 5F**) (HR = 1.050, 95%CI (1.033–1.067), *p* < 0.001) Cox regression, suggesting that the risk score was an independent prognostic factor.

### Potential Signal Axis Identification

**Figure 6:**
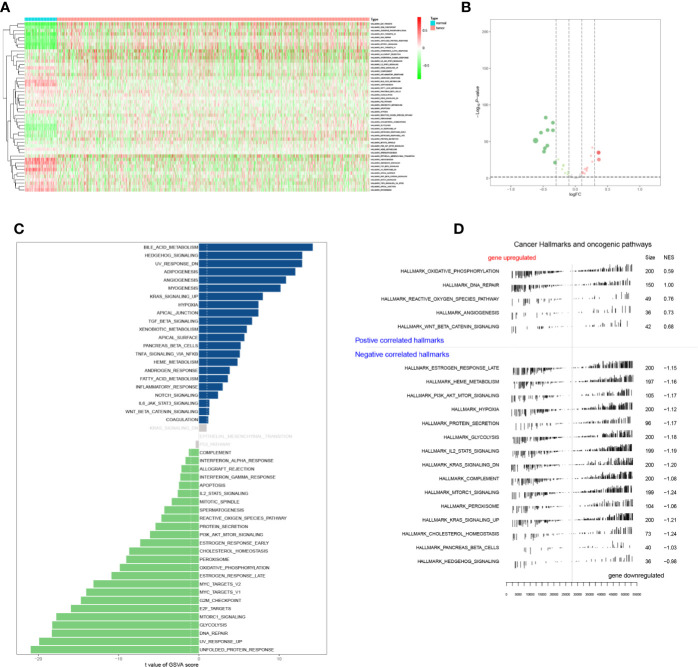
The heatmap **(A)**, volcano plot **(B)**, GSVA **(C)**, and GSEA **(D)** analysis of hallmark gene sets.

The expression levels of Hallmark gene sets were shown in heatmap plot (**Figure 6A**), and differential expressed gene sets showed in volcano plots (**Figure 6B**). A total of 47 significant expressed Hallmark gene sets were identified by GSVA (**Figure 6C**), and five up-regulated and 15 down-regulated Hallmark gene sets were identified by GSEA (**Figure 6D**). What’s more, Hallmark gene sets were co-analyzed with PSRSs by the Pearson correlation analysis.

**Figure 7:**
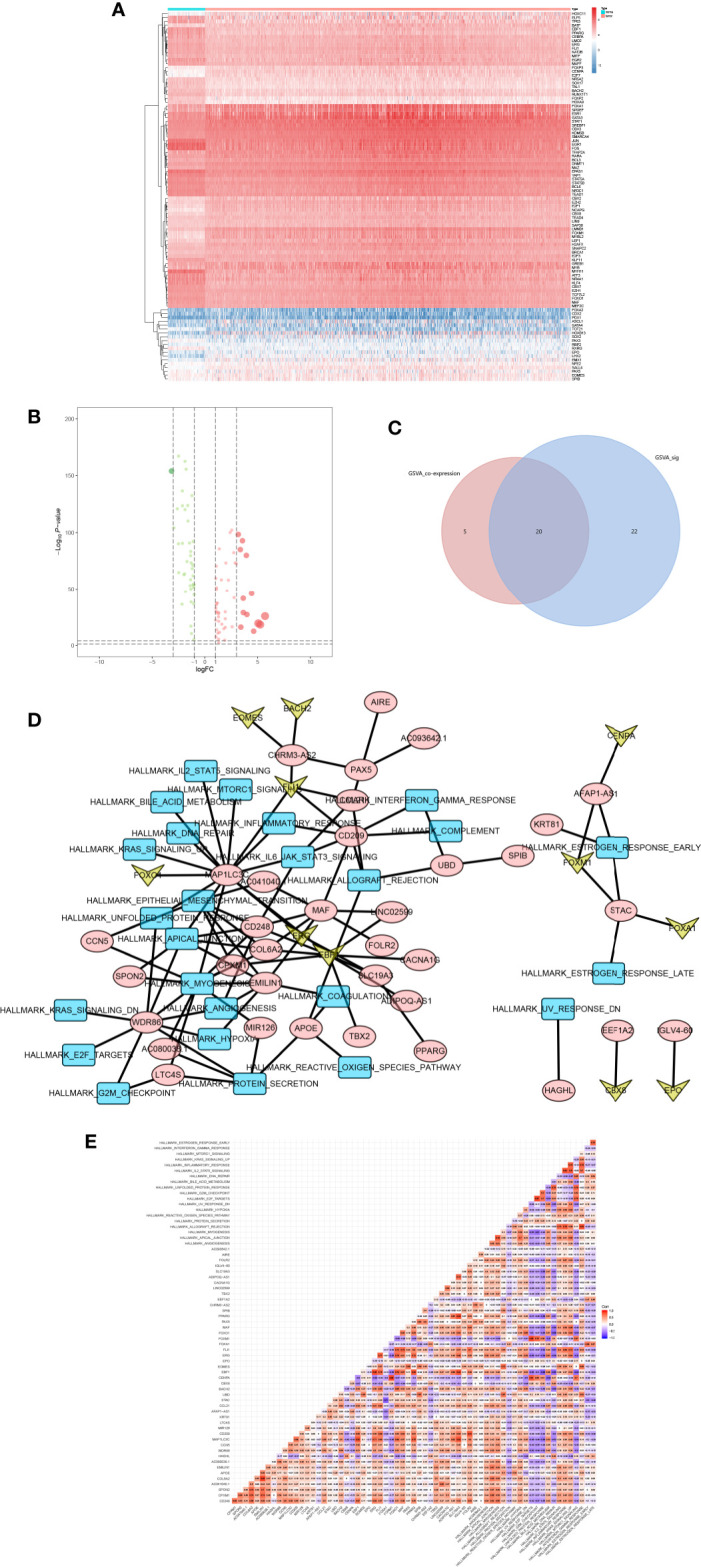
The heatmap **(A)** and volcano plot **(B)** of TFs. And the venn plot of hallmark gene sets **(C)**. The network plot of TFs, DEGs and hallmark gene sets **(D)**. And the co-analysis result for TFs, DEGs and hallmark gene sets **(E)**.

Based on data of 318 TFs from the Cistrome database, a series of analysis on expression launched, and heatmap plot (**Figure 7A**) and volcano plot (**Figure 7B**) were applied. Moreover, 96 TFs were significantly differential expressed. Then, the Pearson correlation analysis was utilized to find the relation between TFs and PSRSs.

In addition, the intersection between co-analysis Hallmark gene sets in GSVA and significant Hallmark gene sets in GSVA was illustrated in Venn plots (**Figure 7C**), 22 Hallmark gene sets were found.

Next, the network (**Figure 7D**) of TFs, PSRSs and Hallmark gene sets were constructed based on the coefficient correlation of the Pearson correlation analysis. Therefore, key TF-PSRS paired was MAF-CD248 (Correlation coefficient = 0.435, *p* < 0.001, positive), and PSRS-Hallmark gene set paired was CD248-apical junction (Correlation coefficient = 0.353, *p* < 0.001) (**Figure 7E**).

In sum, the scientific hypothesis was defined: MAF positively regulated CD248, promoting apical junction pathway in BRCA, which might play a role in bone metastasis.

### Cmap Analysis

**Figure 8:**
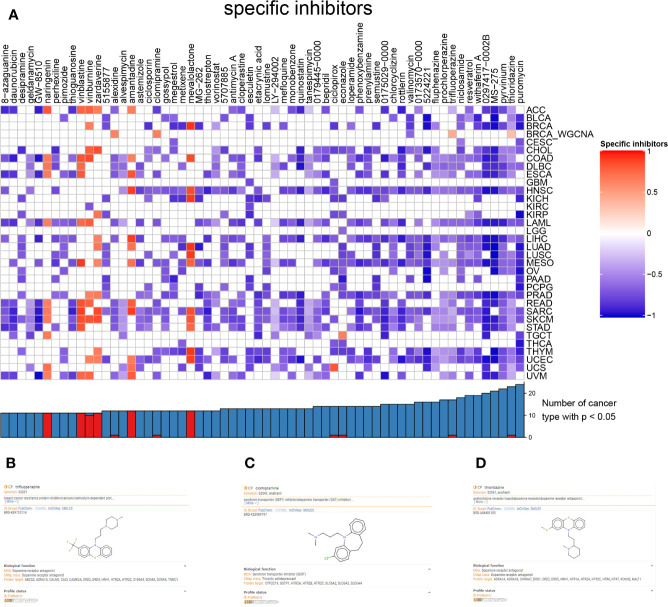
The heatmap of inhibitor and different cancers **(A)**. The information of trifluoperazine **(B)**, clomipramine **(C)**, thioridazine **(D)** from clue database.

To find the latent inhibitor of the bone metastasis-specific regulation network and proposed signal axis, the CMap analysis was utilized, and alexidine (enrichment = 0.6393, *p* = 0.046), clomipramine (enrichment = 0.654, *p* = 0.034), trifluoperazine (enrichment = 0.434, *p* = 0.003), thioridazine (enrichment = 0.337, *p* = 0.016) and valinomycin (enrichment =-0.639, *p* = 0.041) were significant compounds in BRCA (**Figure 8A**). Based on the clue database, the detail information of trifluoperazine (**Figure 8B**), clomipramine (**Figure 8C**) and thioridazine (**Figure 8D**) were found, and trifluoperazine was most related to metastasis BRCA according to literature review results.

### Spatial Transcriptome and Chromatin Immunoprecipitation Sequence Validation

**Figure 9:**
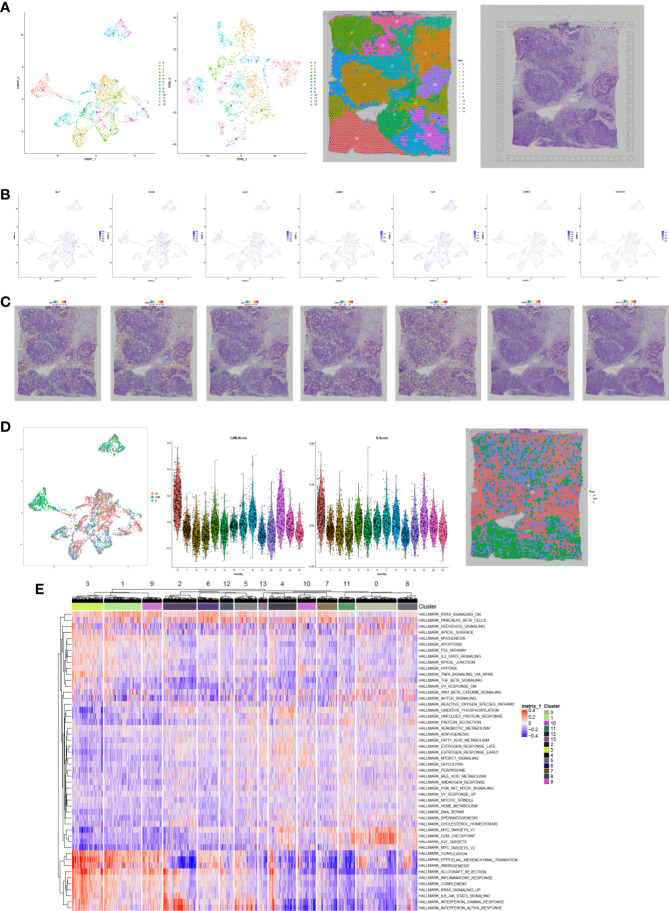
The UMAP, t-SNE plots, and pare-carcinoma, invasive ductal carcinoma and intraductal carcinoma *in situ* were illustrated in HE-stained section **(A)**. The feature and spatial plots of MAF, CD248, GJA1, LAMA3, TJP1, LAMC2, and COL17A1, showing that these key genes were highly expressed in the invasive ductal carcinoma tissue **(B, C)**. UMAP plot related to cell cycle, violin plots of phase G2M and S, and phase annotated section **(D)**, and cluster 4 and 7 of invasive ductal carcinoma and 10 of intraductal carcinoma highly related to phase G2M and S. The heatmap of Hallmark gene sets in cell clusters **(E)**.

**Figure 10:**
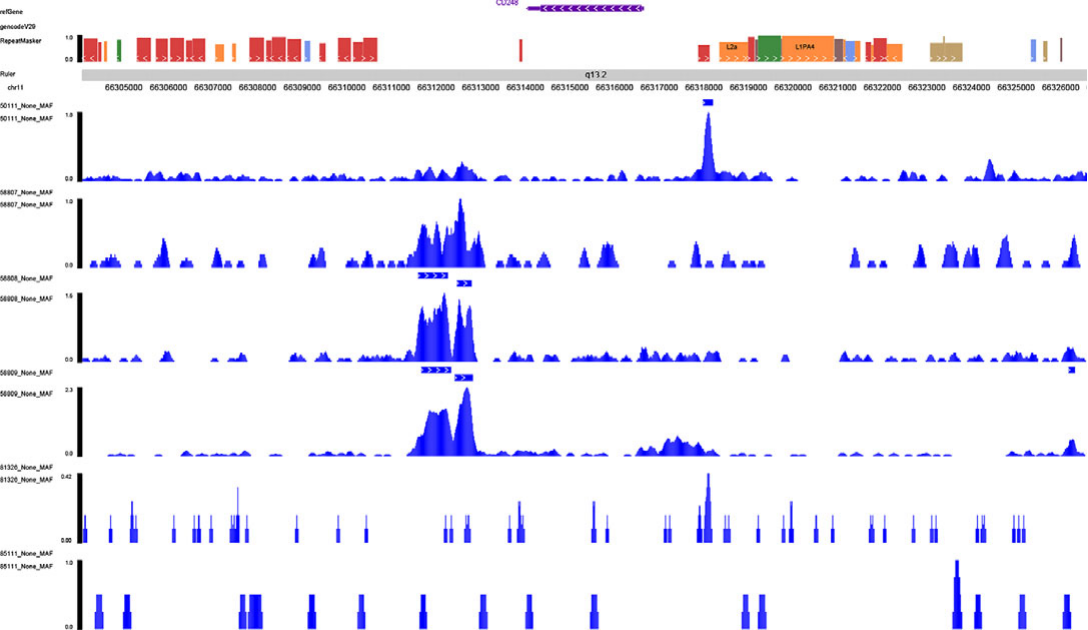
Validation of the transcriptional regulation mechanisms of MAF-CD248 in ChIP-seq data available from Cistrome database.

**Figure 11:**
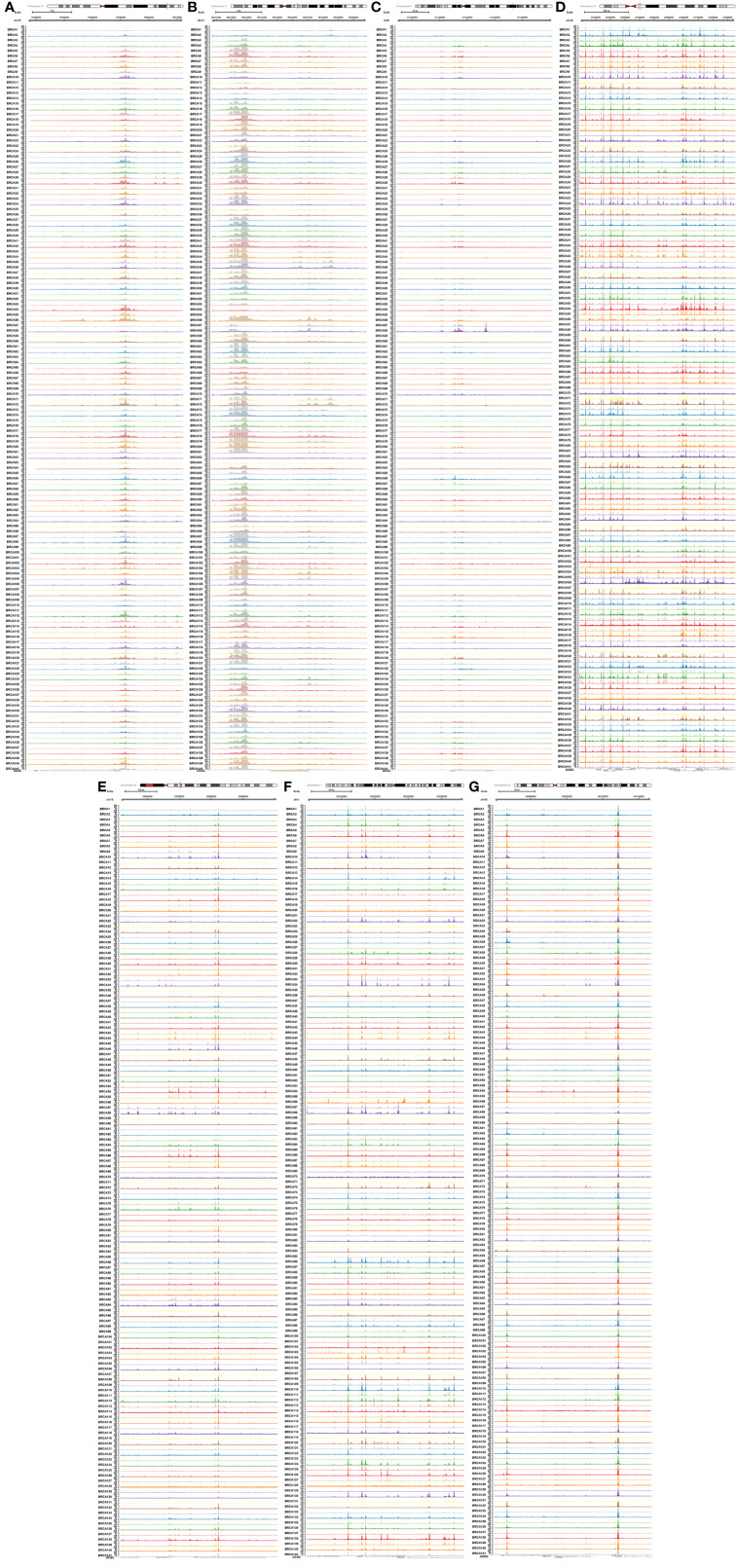
Validation of seven key genes [MAF **(A)**, CD248 **(B)**, GJA1 **(C)**, LAMA3 **(D)**, TJP1 **(E)**, LAMC2 **(F)**, and COL17A1 **(G)**] in ATAC-seq data available from TCGA.

With the aim to further explore the location of key genes in subtype cell clusters, the profiling of scRNA-seq and spatial transcriptome were co-analyzed. Fourteen clusters were identified in UMAP and t-SNE, and pare-carcinoma, invasive ductal carcinoma and intraductal carcinoma *in situ* were illustrated in HE-stained section (**Figure 9A**). For validation, the feature and spatial feature plots of MAF, CD248, GJA1, LAMA3, TJP1, LAMC2, and COL17A1 demonstrated to show the location in BRCA samples, and they were highly-expressed in the invasive ductal carcinoma tissue (cluster 2, 4, 7, 9, 12) (**Figures 9B, C**). Besides, a series of analysis based on cell cycle indicated that genes in cluster 4 and 7 of invasive ductal carcinoma and 10 of intraductal carcinoma highly related to phase G2M and S (**Figure 9D**). In addition, tumor inhibited pathways like apoptosis, p53 pathway and TNFα signaling *via* NF-kB were down-regulated while tumor genesis related pathways G2M checkpoint and E2F targets were up-regulated (**Figure 9E**). In ChIP-seq analysis, binding peaks were illustrated in CD248 sequence (**Figure 10**). Moreover, genes in our hypothesis were validated in ATAC-seq (**Figure 11**), and they were all regulated in BRCA samples. Therefore, patterns of direct transcriptional regulatory between TF-PSRS interaction pairs were identified.

### Multidimensional Validation

The correlation of key genes in signal axis based on cBioportal database was summarized in **Table S1**. And top five genes in apical junction were GJA1, LAMA3, TJP1, LAMC2, and COL17A1. Several databases were applied to validate the expression level (**Table S2**) and prognosis value (**Table S3**) of key genes in hypothesis signal axis. Besides, details were demonstrated in **Figure S1**-**12**. MAF showed down-regulated in BRCA, and CD248, GJA1, LAMA3, TJP1, LAMC2, and COL17A1 showed up-regulated in primary BRCA. Besides, MAF was highly-expressed in the metastasis sample. What’ more, MAF (**Figure S3C**, *p* = 0.013), CD248 (**Figure S3B**, *p* = 0.036), GJA1 (**Figure S3A**, *p* = 0.005; **Figure S6B**, *p* = 0.003), LAMA3 (**Figure S3A**, *p* = 0.008; **Figure S6B**, *p* = 0.006), TPJ1 (**Figure S6B**, *p* = 0.019) and LAMC2 (**Figure S3B**, *p* = 0.018) showed significantly related metastasis; MAF (**Figure S3C**, *p* = 0.002; S6C, *p* = 0.048), CD248 (**Figure S3D**, *p* = 0.021; S6C, *p* = 0.015), GJA1 (**Figure S3E**, *p* = 0.001; S6A, *p* < 0.001), LAMA3 (S6A, *p* = 0.047),TJP1 (**Figure S3D**, *p* = 0.031; **Figure S6A**, *p* = 0.044; **Figure S7A**, *p* = 0.027), LAMC2 (**Figure S5F**, *p* = 0.038) and COL17A1 (**Figure S1R**, *p* = 0.014; **Figure S3D**, *p* = 0.007; **Figure S5G**, *p* = 0.001; **Figure S6A**, *p* = 0.048) showed significantly related prognosis; LAMC2 (**Figure S7C**, *p* = 0.047) and COL17A1 (**Figure S7C**, *p* = 0.001) were significantly related to progression free.

## Discussion

BRCA was a common tumor in female, and patients with bone metastasis suffered from the pain and the risk of fracture and even death (4). What’s worse, tumor genesis and osteolytic damage could be mutually reinforcing (4). As consequence, the mechanism of bone metastasis must be expounded for early diagnosis and precise therapy.

In the recent study, a total of 813 primary BRCA samples were analyzed. Based on WGCNA method and univariate Cox regression analysis, several modules were annotated by mRNAsi and Hallmark gene sets to find the key module and correspond PSRSs. And the multivariate Cox model was constructed. In addition, multivariate Cox model and risk score were accessed by ROC curve and Kaplan-Meier survival analysis. And the risk score was an independent predict factor. Then, a metastasis-specific regulation network was constructed by the Pearson analysis, and MAF, CD248 and apical junction were significant. Moreover, the regulatory pattern was supported by spatial single-cell RNA sequence and ChIP-seq data and multi-omics online databases. Based on CMap analysis, trifluoperazine was identified as the possible inhibitor for bone metastasis of BRCA.

MAF was MAF basic leucine zipper transcription factor, which belonged to AP-1 super family, and it regulated the terminal differentiation (34). Besides, MAF was crucial in promoting osteoblast differentiation of bone marrow stromal cell (35). What’ more, MAF directly regulated osteoblast-specifical promoter Bglap1, and co-regulated the osteoblast differentiation with RUNX2 (35). In addition, Milica Pavlovic et al. found that high-expressed MAF was significant related to bone metastasis instead of visceral metastasis based on Genomic copy number distortion analysis and immunohistochemistry, and MAF was also correlated to overall survival (36). Moreover, MAF played a role in promoting bone metastasis rather than cancer cell proliferation *in vivo* (36), which was consistent with our scientific hypothesis. Additionally, MAF also potentially controlled biological processes like migration, adhesion, and osteoclast differentiation in bone metastasis (36).

CD248 highly-expressed in tumor tissue, especially in BRCA, and it was related to the prognosis of patients (37). And the transcription product of CD248 was endosialin, which expressed on the cell surface of fibroblasts and pericytes in tumor instead of tumor endothelium (38). Further, Carmen Viski et al. found CD248 played a pivotal role by promoting the step of infiltration from primary to circulatory system *via* pericytes in metastasis, which affected on tumor microenvironment (39). Besides, CD248 enhanced the adhesion to the extracellular matrix, and activated the matrix metalloproteinase 9 (MMP9) in tumor metastasis (40). Moreover, CD248 expressed in osteoblast instead of osteoclast, and it has negative effects on osteoblast maturation and ossification (41).

Although none of the study reported the correlation of MAF and CD248, we proposed that MAF positively regulated the transcription of CD248: promoted the function of the transcript in cell adhesion, invasion and migration, and regulated osteoblast function (36, 40, 41).

Apical junction was a structure of apical domain of epithelial cells, and it linked adjacent epithelial cells by tight and adherent junction, which was essential for maintaining the epithelial barrier (42). And in type III epithelial–mesenchymal transition (EMT), the apical junction was disrupted, the apical–basal polarity lost, and the mesenchymal characteristics emerged, finally cells migrated to vessels and traveled to multiply organs and tissues (43). However, some epithelial characteristics were retained, especially the E-cadherin, and the collective migration was observed in metastasis BRCA (44, 45). What’s more, when in the stage of transplanting to the bone, E-cadherin and E-cadherin adherent junction formed between cancer cells, then the E-cadherin and N-cadherin adherent junction formed between cancer cells and osteoblasts, and cell-cell contact promoted the tumor proliferation *via* activated mTOR pathway in tumor microenvironment (46).

Up to date, few of study focused on the relationship between CD248 and apical junction. We speculated that CD248 regulated the apical junction by promoting the bone colonization in bone metastasis (41, 46).

Trifluoperazine was a type of calmodulin blocker and dopamine D2 like receptor antagonist, which could inhibit the cancer cell metastasis *in vitro*, but the collective migration can be promoted by knocking out MRP (47). However, trifluoperazine might disturb the electrostatic surface potential (47). Besides, it inhibited the differentiation of osteoclasts, tumor genesis, metastasis and bone loss and promoted bone formation in breast cancer (48). Therefore, we proposed trifluoperazine might target on the MAF-CD248-apical junction axis, and play a role in inhibiting metastasis.

Last but not least, there were some limitations in our study. Firstly, our analysis was only based on high-throughput bioinformatic analysis rather than mechanism exploration. Then, data and platform were also limited, and more validations based on different data sets were needed (e.g. most ChIP-seq samples were not BRCA). And verifications based on clinical samples (with and without metastasis) were required. Next, more function experiments and directly mechanism needed to be verified. Therefore, gain/loss experiments based on MAF-CD248, CD248-apical junction and MAF-apical junction, rescue experiments on MAF-CD248-apical junction axis *in vivo* and vitro and Co-Immunoprecipitation will be lunched. Importantly, the MAF-CD248-apical junction axis in bone metastasis BRCA was firstly reported, and our study provided the idea of application on clinical prognosis and precise therapy.

## Conclusion

In summary, we proposed that MAF positively regulated CD248, then promoted apical junction pathway in BRCA, which played a role in bone metastasis. And it could be inhibited by trifluoperazine. Besides, the MAF-CD248-apical junction signal axis was verified by spatial single-cell RNA sequence and ChIP-seq data and multi-omics online databases.

## Data Availability Statement

Publicly available datasets were analyzed in this study. This data can be found here: TCGA program (https://portal.gdc.cancer.gov) and 10x genomics support center (https://support.10xgenomics.com/spatial-gene-expression/datasets).

## Ethics Statement

The study was approved by the Ethics Committee of the First Affiliated Hospital of Zhengzhou University. The patients/participants provided their written informed consent to participate in this study.

## Author Contributions

RH, ZL, JiayZ, ZZ, JiaqZ, ML, SW, SX, YX, XC, JL, WC, BW, PY, DY, and ZH conceived and designed the study. RH, ZL, JiayZ, ZZ, JiaqZ, ML, SW, SX, YX, XC, JL, WC, BW, PY, DY, and ZH collected and/or assembled the data. RH, ZL, JiayZ, ZZ, JiaqZ, ML, SW, SX, YX, XC, JL, WC, BW, PY, DY, and ZH analyzed and interpreted the data. RH, ZL, JiayZ, ZZ, JiaqZ, ML, SW, SX, YX, XC, JL, WC, BW, PY, DY, and ZH wrote the manuscript. RH, ZL, JiayZ, ZZ, Jiaq, ML, SW, SX, YX, XC, JL, WC, BW, PY, DY, and ZH gave the final approval of the manuscript. All authors contributed to the article and approved the submitted version.

## Funding

The study was supported by the National Natural Science Foundation of China, joint fund cultivation project (Grant No. U1504822); Henan Medical Science and Technology Research Project (No. 201602031); Henan Medical Science and technology Research Plan, Joint Project of the Ministry and the Province, (No. SB201901037); Henan Provincial Department of Science and Technology, Social Development Project (No. 142102310055). The funders had no role in study design, data collection and analysis, decision to publish, or preparation of the manuscript.

## Conflict of Interest

The authors declare that the research was conducted in the absence of any commercial or financial relationships that could be construed as a potential conflict of interest.

## Supplementary Material

Supplementary Figure 1 The expression level of MAF **(A)**, CD248 **(B)**, GJA1 **(C)**, LAMA3 **(D)**, TJP1 **(E)**, LAMC2 **(F)**, and COL17A1 **(G)** between tumor and normal. The stage plot of MAF **(H)**, CD248 **(I)**, GJA1 **(J)**, LAMA3 **(K)**, TJP1 **(L)**, LAMC2 **(M)**, and COL17A1 **(N)** in BRCA. The Kaplan-Meier survival analysis for overall survival in MAF **(O)**, CD248 **(P)**, LAMA3 **(Q)**, and COL17A1 **(R)**, for Disease free survival in MAF **(S)**, CD248 **(T)**, LAMA3 **(U)**, and COL17A1 **(V)**.

Supplementary Figure 2 The expression level of MAF **(A)**, CD248 **(B)**, GJA1 **(C)**, LAMA3 **(D)**, TJP1 **(E)**, LAMC2 **(F)**, and COL17A1 **(G)** between tumor and normal.

Supplementary Figure 3 The Kaplan-Meier survival analysis for MAF, GJA1, LAMA3, TJP1, LAMC2, COL17A1, and integrated genes in BRCA metastasis in NKI **(A)**; MAF, CD248, GJA1, LAMA3, TJP1, LAMC2, COL17A1, and integrated genes in BRCA metastasis in GSE2990 **(B)**; MAF, CD248, GJA1, LAMA3, TJP1, LAMC2, COL17A1, and integrated genes in BRCA metastasis in GSE11121 **(C)**. The Kaplan-Meier survival analysis for MAF, CD248, GJA1, LAMA3, TJP1, LAMC2, COL17A1, and integrated genes in BRCA overall survival in GSE19783 **(D)**; MAF, CD248, GJA1, LAMA3, TJP1, LAMC2, COL17A1, and integrated genes in BRCA overall survival in GSE3494 **(E)**.

Supplementary Figure 4 The expression level of MAF **(A)**, CD248 **(B)**, GJA1 **(C)**, LAMA3 **(D)**, TJP1 **(E)**, LAMC2 **(F)**, and COL17A1 **(G)** between tumor and normal.

Supplementary Figure 5 The Kaplan-Meier survival analysis of MAF **(A)**, CD248 **(B)**, GJA1 **(C)**, LAMA3 **(D)**, TJP1 **(E)**, LAMC2 **(F)**, and COL17A1 **(G)** between high- and low-expression groups. The positively **(H)** and negatively **(I)** related genes of BRCA.

Supplementary Figure 6 The Kaplan-Meier survival analysis for MAF, CD248, GJA1, LAMA3, TJP1, LAMC2, COL17A1, and integrated genes in BRCA overall survival in Van **(A)**; MAF, CD248, GJA1, LAMA3, TJP1, LAMC2, COL17A1, and integrated genes in BRCA metastasis in Van **(B)**; MAF, CD248, GJA1, LAMA3, TJP1, LAMC2, COL17A1, and integrated genes in BRCA overall survival in TCGA **(C)**.

Supplementary Figure 7 The Kaplan-Meier survival analysis for MAF, CD248, GJA1, LAMA3, TJP1, LAMC2, COL17A1, and integrated genes in BRCA overall survival **(A)**; MAF, CD248, GJA1, LAMA3, TJP1, LAMC2, COL17A1, and integrated genes in BRCA disease free **(B)**; MAF, CD248, GJA1, LAMA3, TJP1, LAMC2, COL17A1, and integrated genes in BRCA progression free **(C)**.

Supplementary Figure 8 The expression level of MAF, CD248, GJA1, LAMA3, TJP1, LAMC2, and COL17A1 in normal people tissue.

Supplementary Figure 9 The expression level of MAF, CD248, GJA1, LAMA3, TJP1, LAMC2, and COL17A1 in BRCA **(A)**. The PCA plot of MAF, CD248, GJA1, LAMA3, TJP1, LAMC2, and COL17A1 **(B)**. The heatmap of MAF, CD248, GJA1, LAMA3, TJP1, LAMC2, and COL17A1 **(C)**.

Supplementary Figure 10 The expression level of MAF **(A)**, CD248 **(B)**, GJA1 **(C)**, LAMA3 **(D)**, TJP1 **(E)**, LAMC2 **(F)**, and COL17A1 **(G)** in cancer cell lines.

Supplementary Figure 11 The expression level of MAF **(A)**, CD248 **(B)**, GJA1 **(C)**, LAMA3 **(D)**, TJP1 **(E)**, LAMC2 **(F)**, and COL17A1 **(G)** in normal and BRCA tissue.

Supplementary Figure 12 The protein-protein interaction network of MAF, CD248, GJA1, LAMA3, TJP1, LAMC2, and COL17A1.

## Abbreviations

BRCA, Breast invasive carcinoma; ACS, American Cancer Society; TF, Transcription Factor; TCGA, The Cancer Genome Atlas; mRNAsi, mRNA stemness index; PSRS, prognostic stemness-related signature; KEGG, Kyoto Encyclopedia of Genes and Genomes; GSEA, Gene-Set Enrichment Analysis; GSVA, Gene Set Variation Analysis; CCLE, Cancer Cell Line Encyclopedia; GEPIA, Gene Expression Profiling Interactive Analysis; GTEx, Genotype-Tissue Expression; ROC, The Receiver Operator Characteristic; AUC, Area Under the Curve; FDR, False Discovery Rate; GO, Gene Oncology; KEGG, Kyoto Encyclopedia of Genes and Genomes; DEG, Differential Expressed Gene; FC, Fold charge; mRNAsi, mRNA Expression-Based Stemness Index; GS, gene significance; MS, module significance; ME, module eigengenes; MM, module membership; PCA, principal component analysis; PC, principal component; t-SNE, t-distributed Stochastic Neighbor Embedding; UMAP, Uniform Manifold Approximation and Projection; HE, Haematoxylin and Eosin; MMP9, matrix metalloproteinase 9; EMT, epithelial–mesenchymal transition.
