# Supplementary figures and images for: Construction of Bone Metastasis-Specific Regulation Network Based on Prognostic Stemness-Related Signatures in Breast Invasive Carcinoma
Source: Front Oncol. 2021 Jan 27;10:613333. doi: 10.3389/fonc.2020.613333 (PMC7875018; doi:10.3389/fonc.2020.613333)

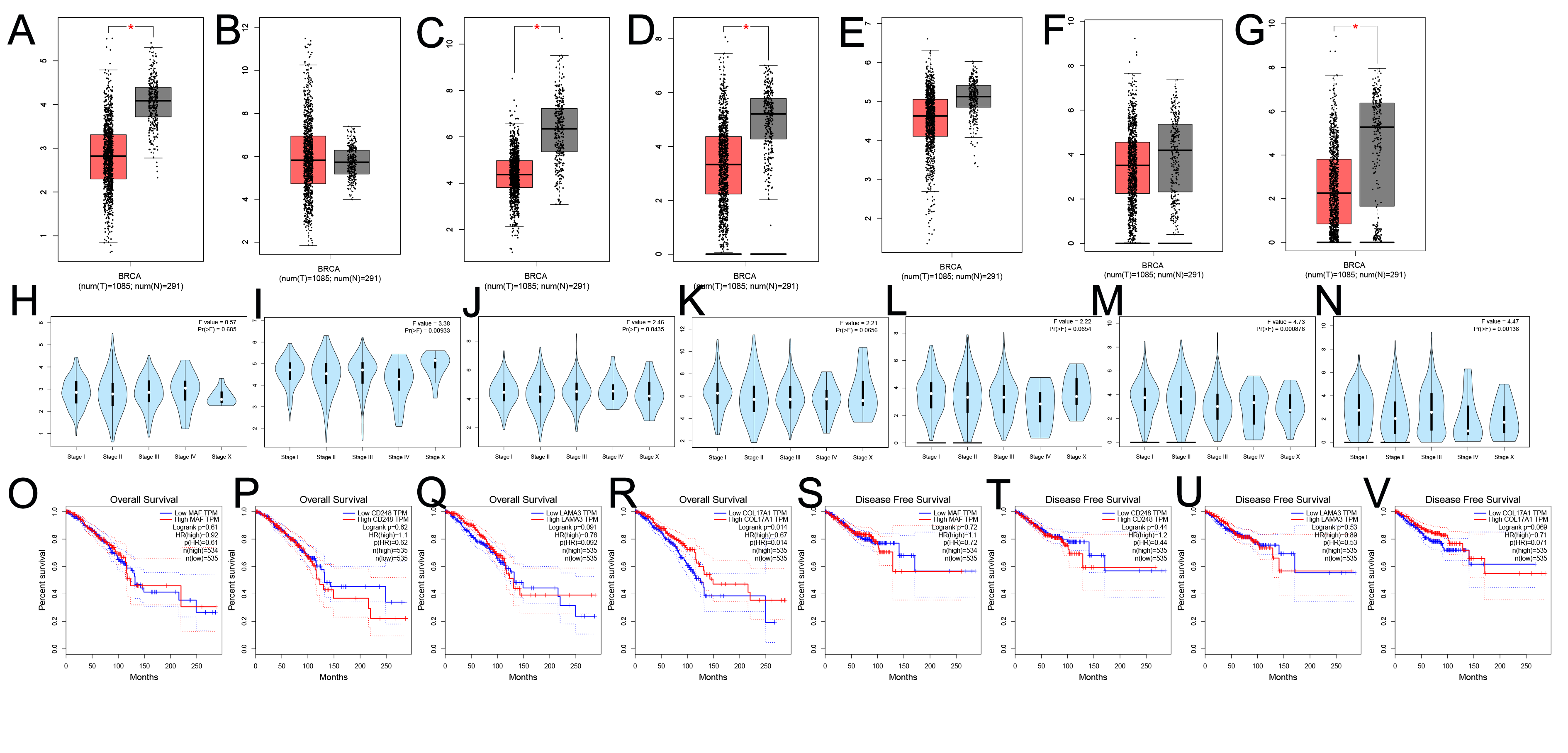

Supplement: Supplementary Figure 1 — The expression level of MAF (A), CD248 (B), GJA1 (C), LAMA3 (D), TJP1 (E), LAMC2 (F), and COL17A1 (G) between tumor and normal. The stage plot of MAF (H), CD248 (I), GJA1 (J), LAMA3 (K), TJP1 (L), LAMC2 (M), and COL17A1 (N) in BRCA. The Kaplan-Meier survival analysis for overall survival in MAF (O), CD248 (P), LAMA3 (Q), and COL17A1 (R), for Disease free survival in MAF (S), CD248 (T), LAMA3 (U), and COL17A1 (V). [file Image_1.tif]

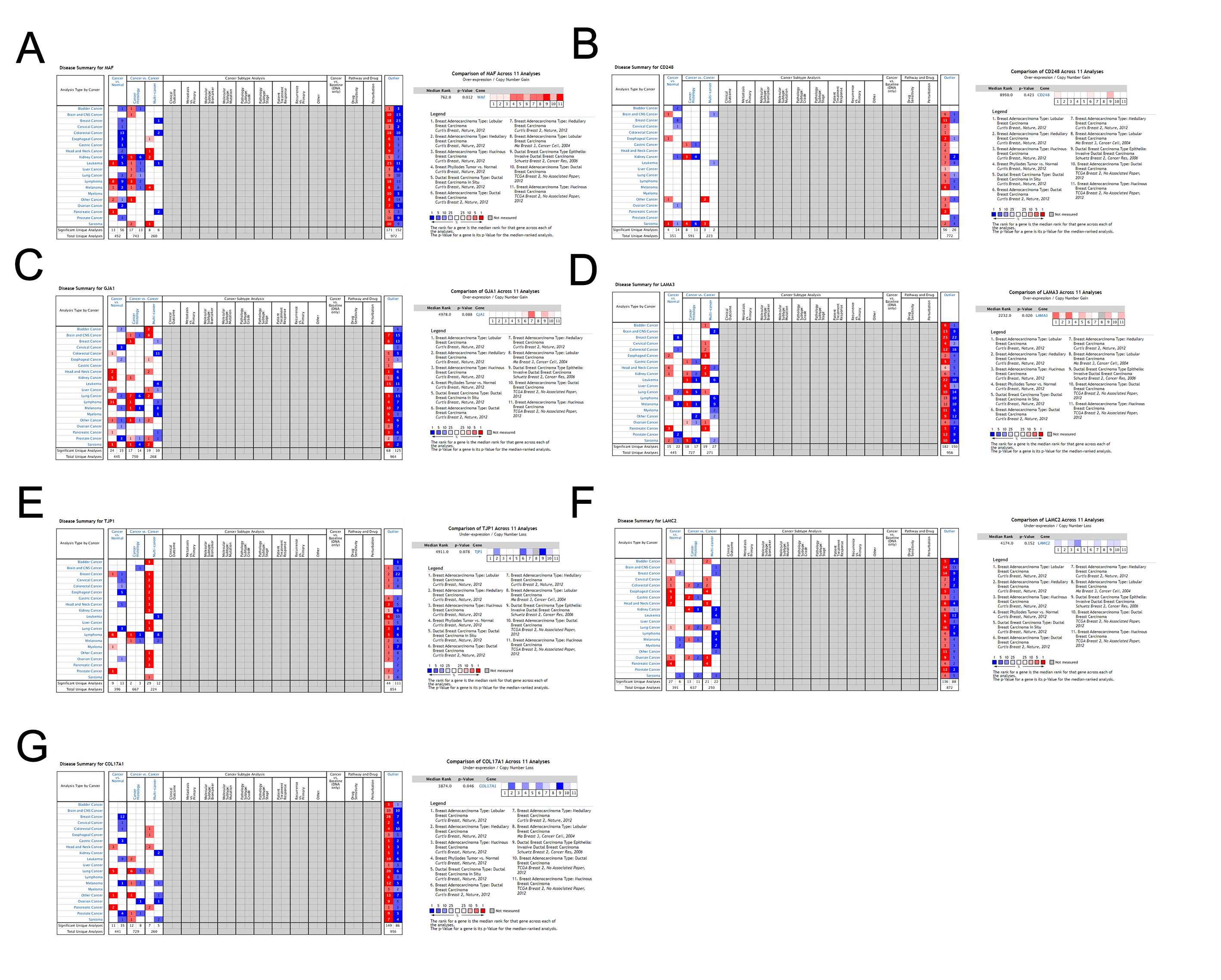

Supplement: Supplementary Figure 2 — The expression level of MAF (A), CD248 (B), GJA1 (C), LAMA3 (D), TJP1 (E), LAMC2 (F), and COL17A1 (G) between tumor and normal. [file Image_2.tif]

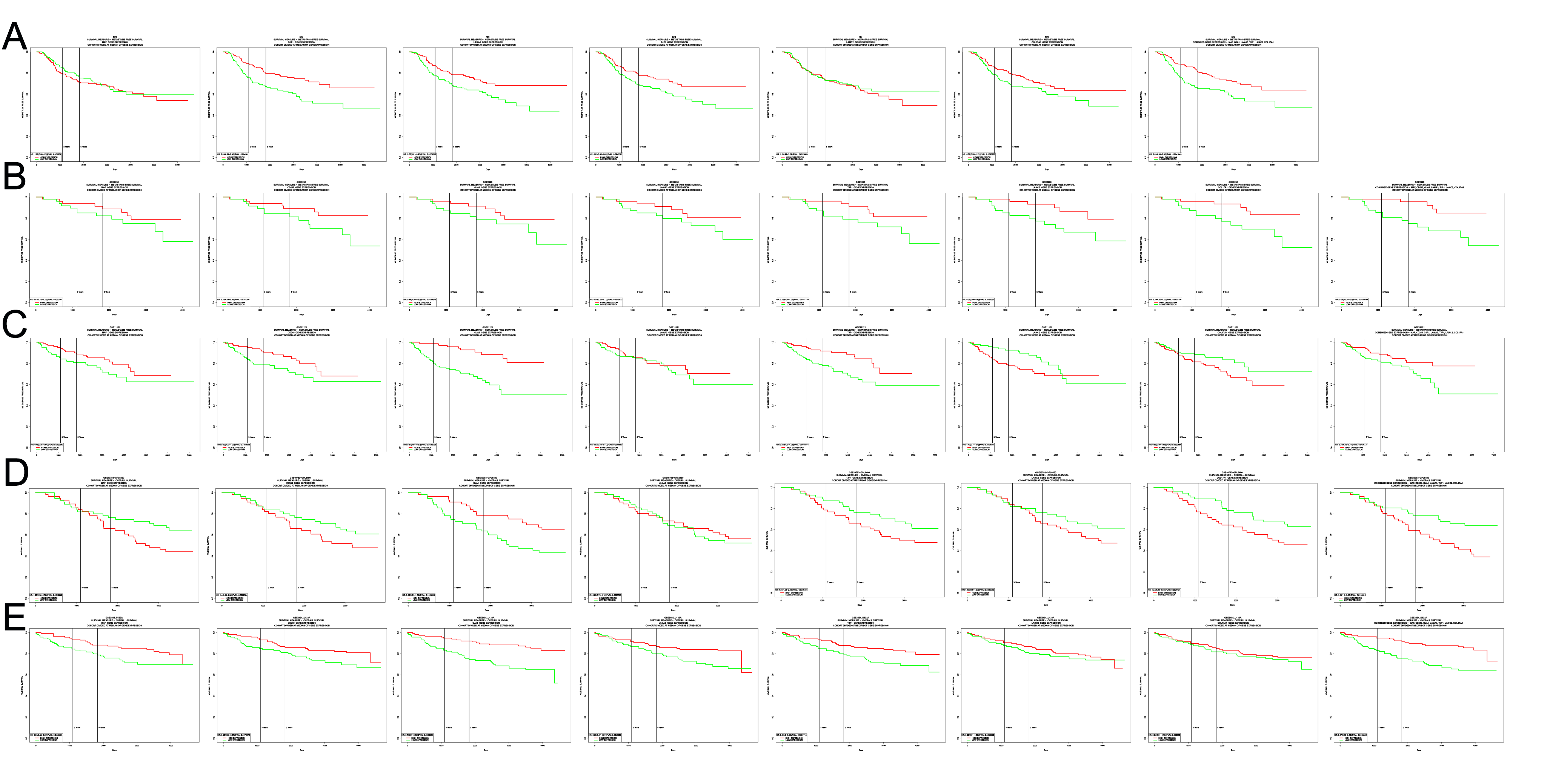

Supplement: Supplementary Figure 3 — The Kaplan-Meier survival analysis for MAF, GJA1, LAMA3, TJP1, LAMC2, COL17A1, and integrated genes in BRCA metastasis in NKI (A); MAF, CD248, GJA1, LAMA3, TJP1, LAMC2, COL17A1, and integrated genes in BRCA metastasis in GSE2990 (B); MAF, CD248, GJA1, LAMA3, TJP1, LAMC2, COL17A1, and integrated genes in BRCA metastasis in GSE11121 (C). The Kaplan-Meier survival analysis for MAF, CD248, GJA1, LAMA3, TJP1, LAMC2, COL17A1, and integrated genes in BRCA overall survival in GSE19783 (D); MAF, CD248, GJA1, LAMA3, TJP1, LAMC2, COL17A1, and integrated genes in BRCA overall survival in GSE3494 (E). [file Image_3.tif]

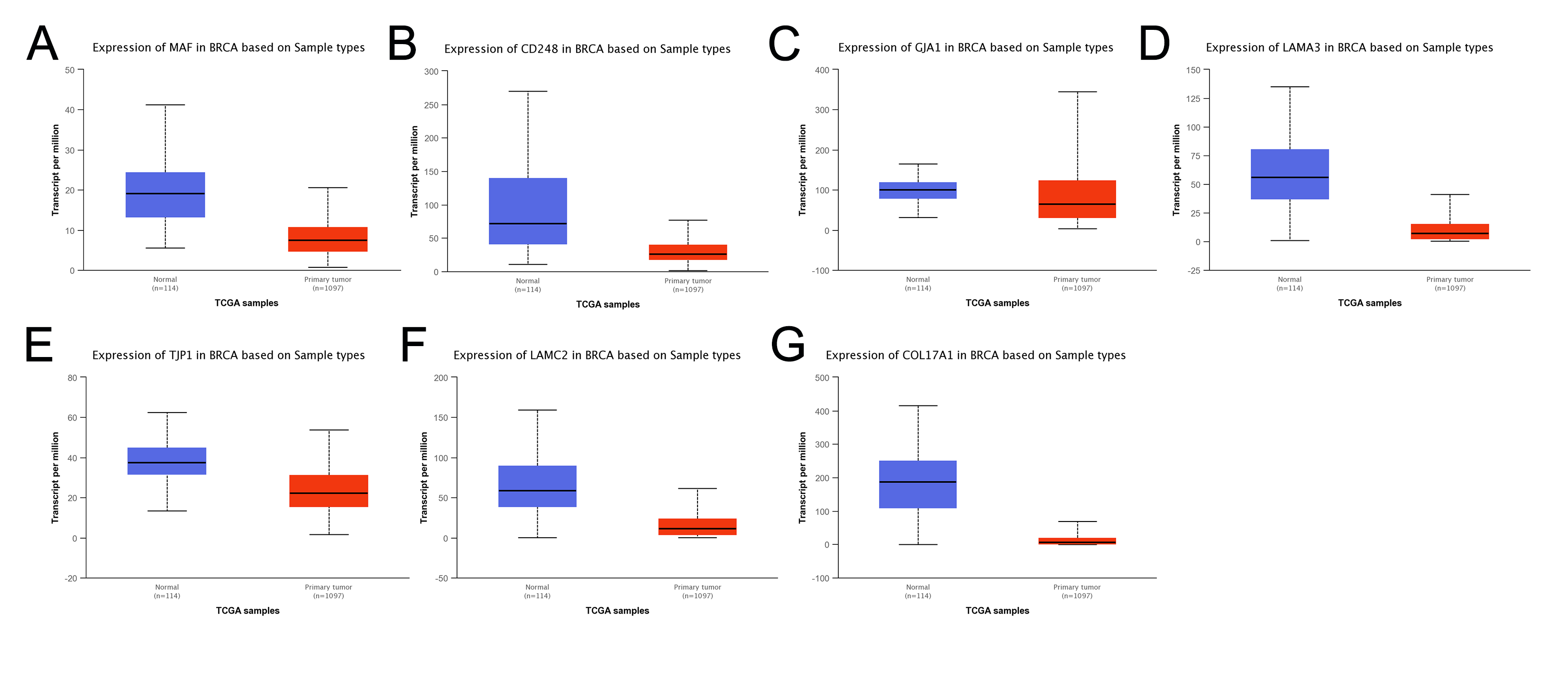

Supplement: Supplementary Figure 4 — The expression level of MAF (A), CD248 (B), GJA1 (C), LAMA3 (D), TJP1 (E), LAMC2 (F), and COL17A1 (G) between tumor and normal. [file Image_4.tif]

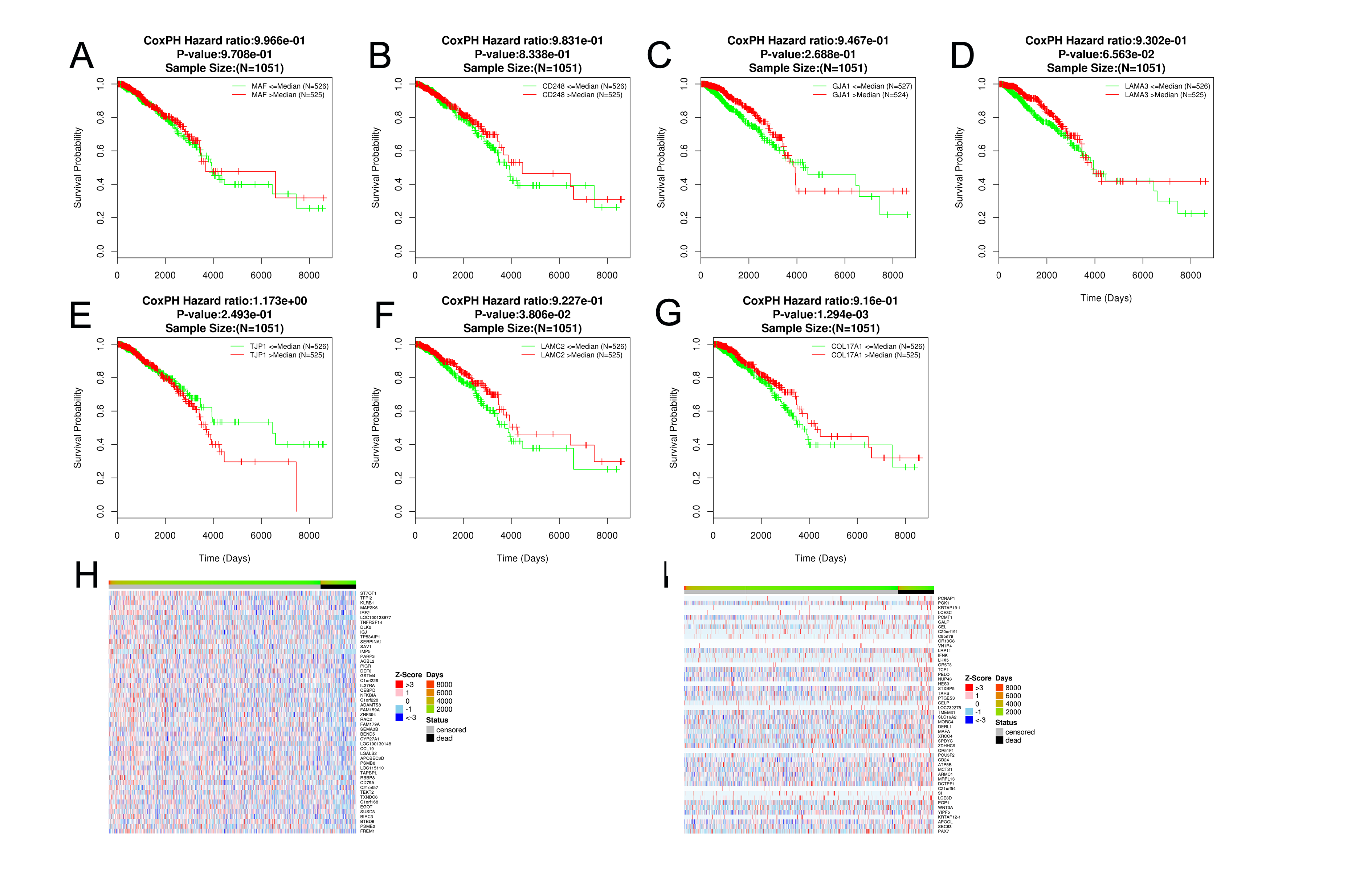

Supplement: Supplementary Figure 5 — The Kaplan-Meier survival analysis of MAF (A), CD248 (B), GJA1 (C), LAMA3 (D), TJP1 (E), LAMC2 (F), and COL17A1 (G) between high- and low-expression groups. The positively (H) and negatively (I) related genes of BRCA. [file Image_5.tif]

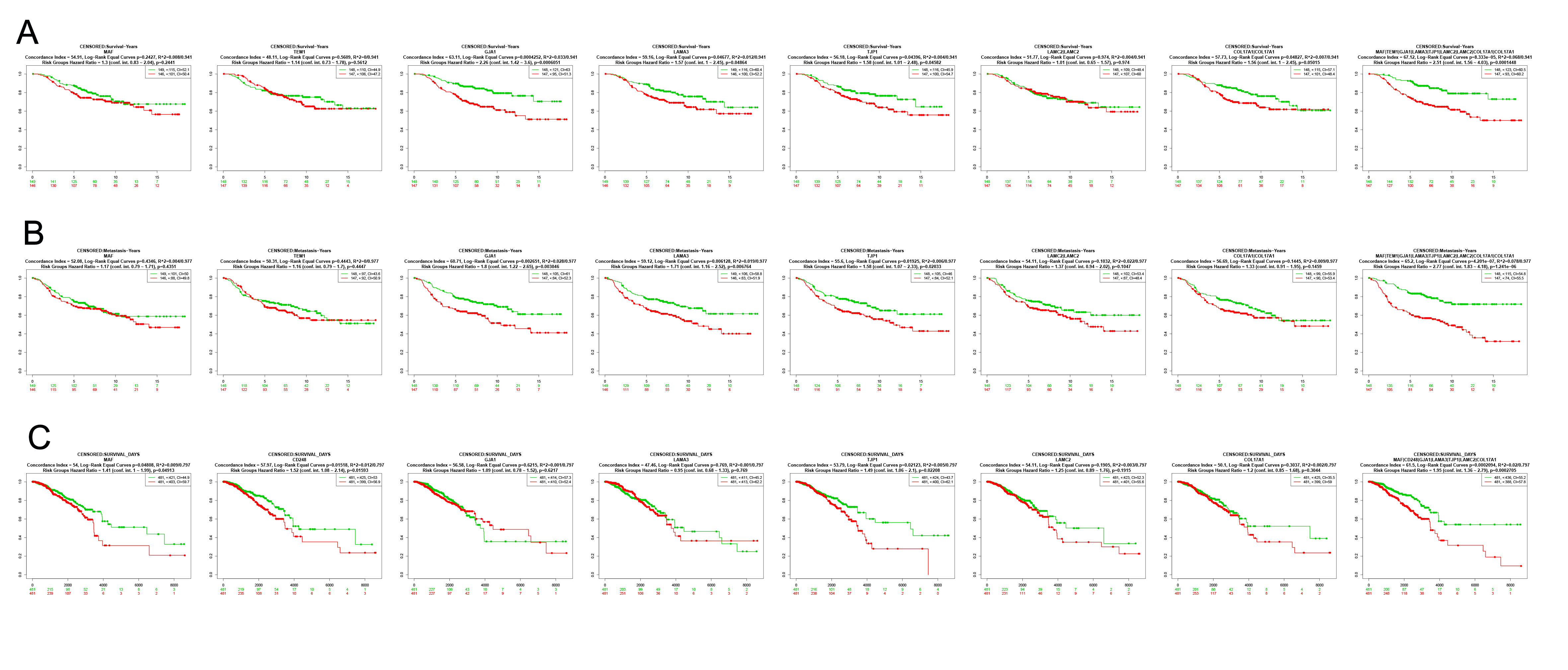

Supplement: Supplementary Figure 6 — The Kaplan-Meier survival analysis for MAF, CD248, GJA1, LAMA3, TJP1, LAMC2, COL17A1, and integrated genes in BRCA overall survival in Van (A); MAF, CD248, GJA1, LAMA3, TJP1, LAMC2, COL17A1, and integrated genes in BRCA metastasis in Van (B); MAF, CD248, GJA1, LAMA3, TJP1, LAMC2, COL17A1, and integrated genes in BRCA overall survival in TCGA (C). [file Image_6.tif]

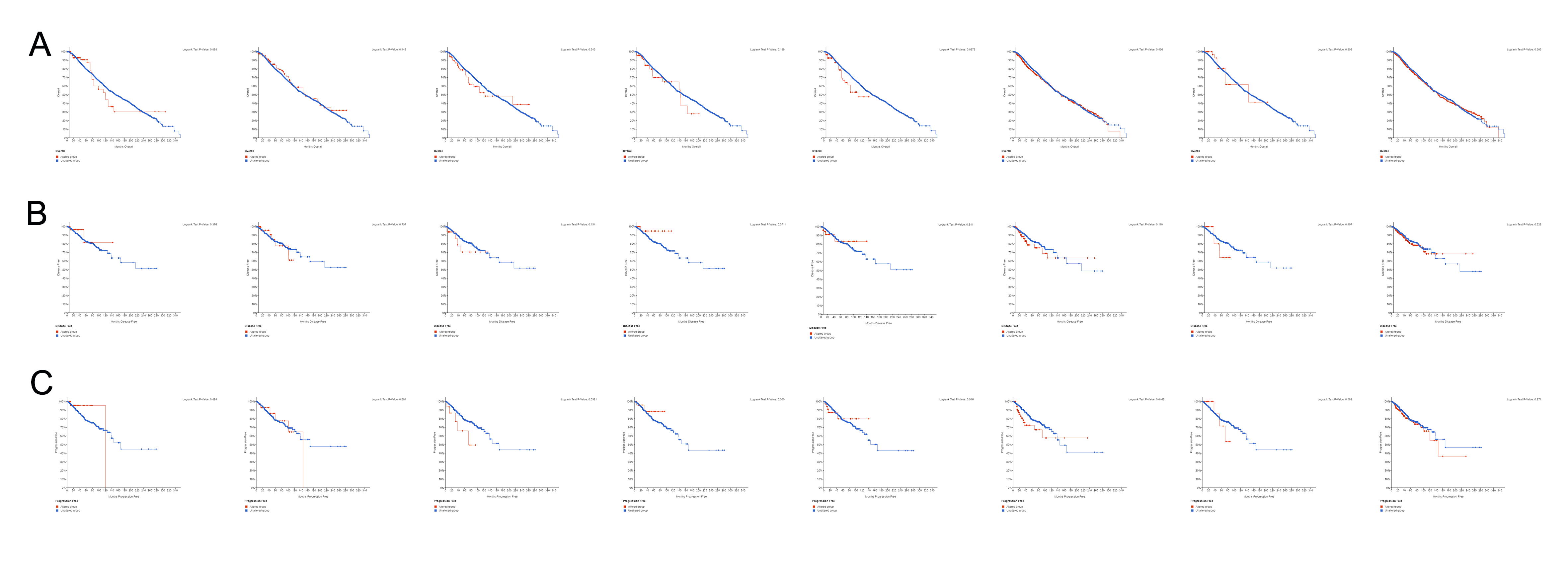

Supplement: Supplementary Figure 7 — The Kaplan-Meier survival analysis for MAF, CD248, GJA1, LAMA3, TJP1, LAMC2, COL17A1, and integrated genes in BRCA overall survival (A); MAF, CD248, GJA1, LAMA3, TJP1, LAMC2, COL17A1, and integrated genes in BRCA disease free (B); MAF, CD248, GJA1, LAMA3, TJP1, LAMC2, COL17A1, and integrated genes in BRCA progression free (C). [file Image_7.tif]

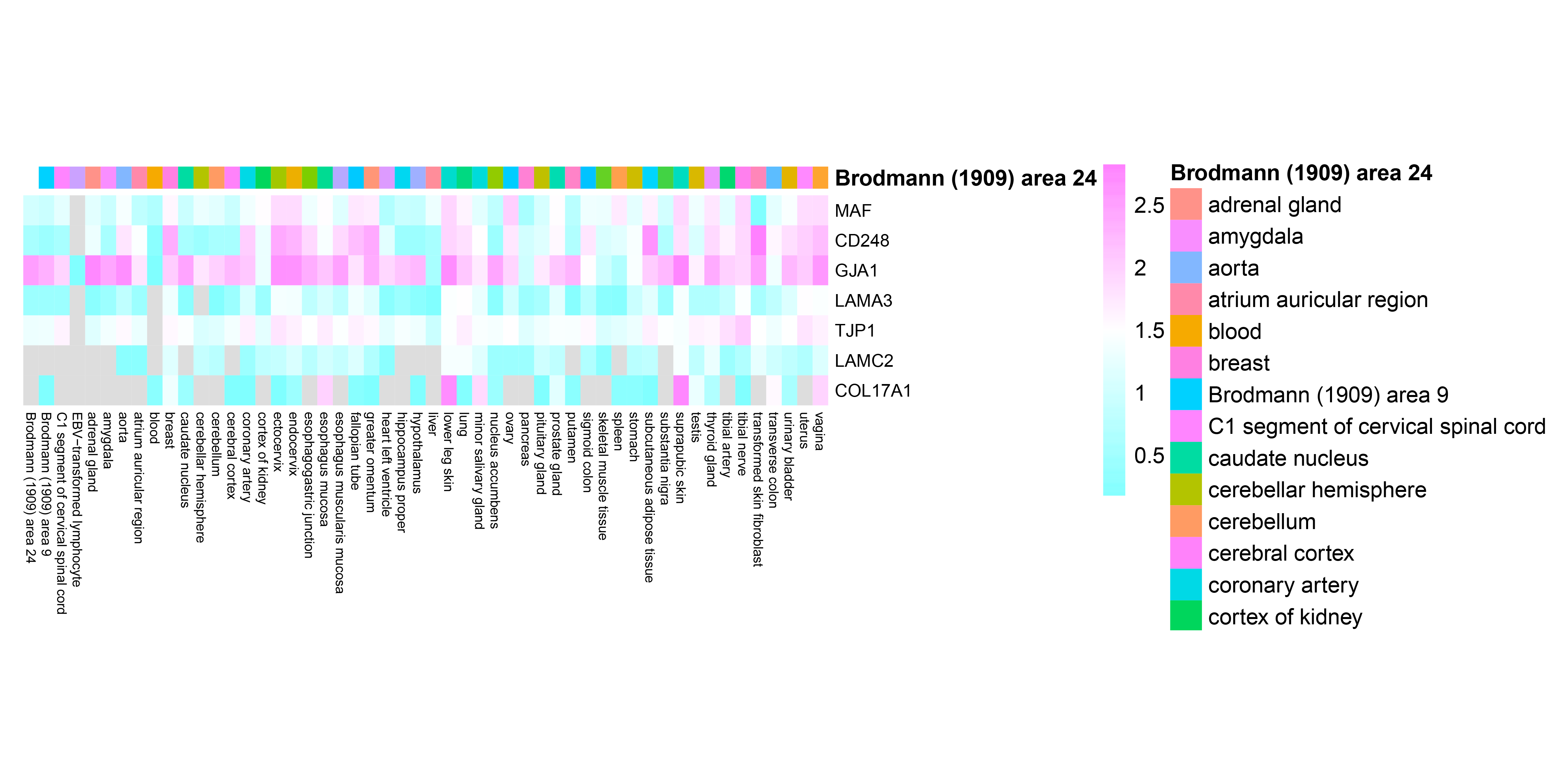

Supplement: Supplementary Figure 8 — The expression level of MAF, CD248, GJA1, LAMA3, TJP1, LAMC2, and COL17A1 in normal people tissue. [file Image_8.tif]

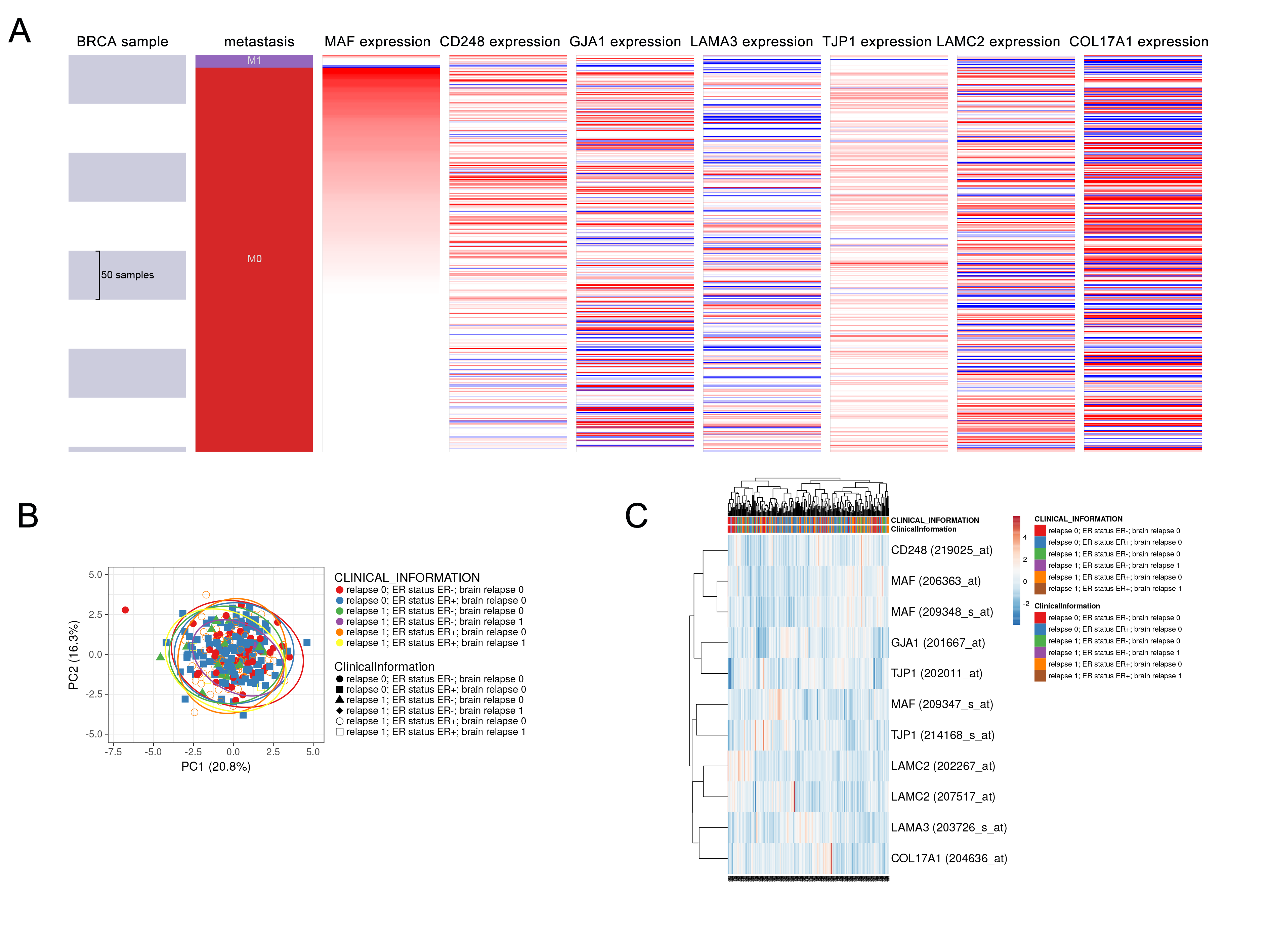

Supplement: Supplementary Figure 9 — The expression level of MAF, CD248, GJA1, LAMA3, TJP1, LAMC2, and COL17A1 in BRCA (A). The PCA plot of MAF, CD248, GJA1, LAMA3, TJP1, LAMC2, and COL17A1 (B). The heatmap of MAF, CD248, GJA1, LAMA3, TJP1, LAMC2, and COL17A1 (C). [file Image_9.tif]

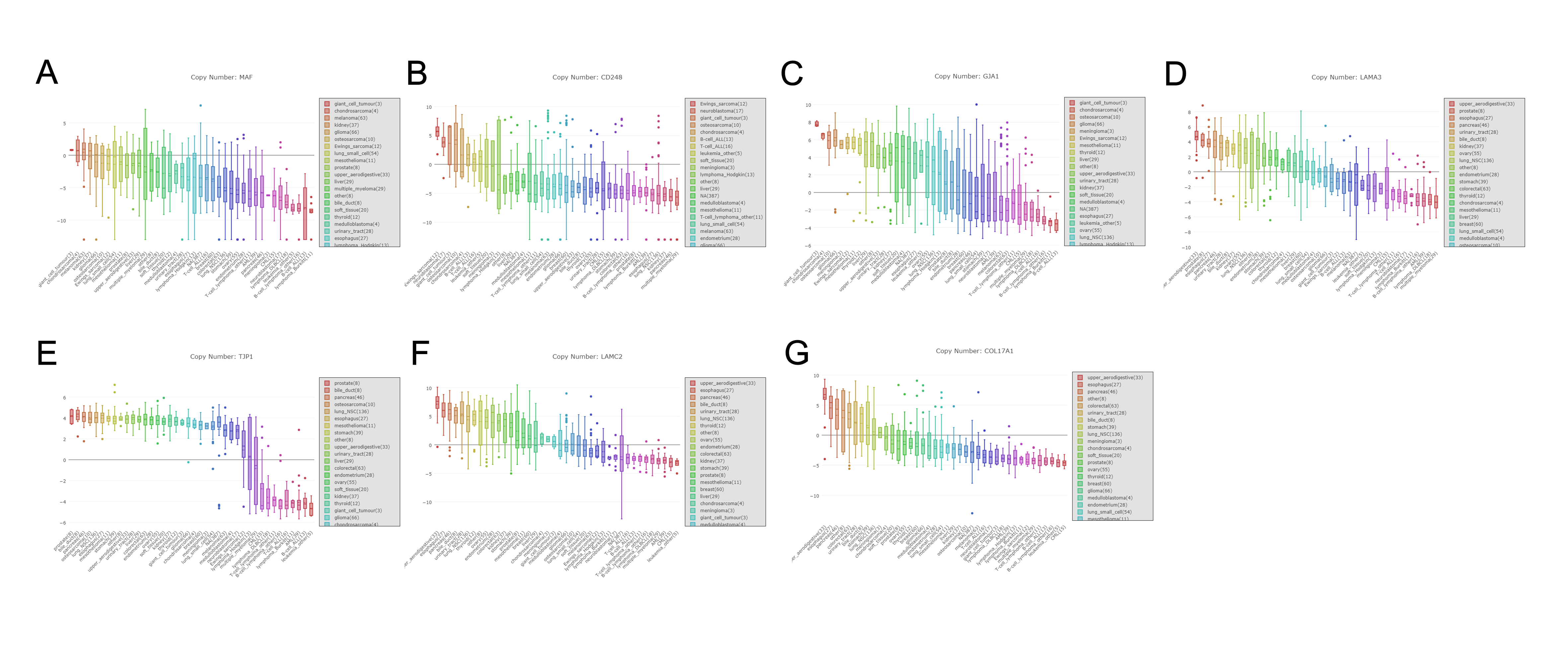

Supplement: Supplementary Figure 10 — The expression level of MAF (A), CD248 (B), GJA1 (C), LAMA3 (D), TJP1 (E), LAMC2 (F), and COL17A1 (G) in cancer cell lines. [file Image_10.tif]

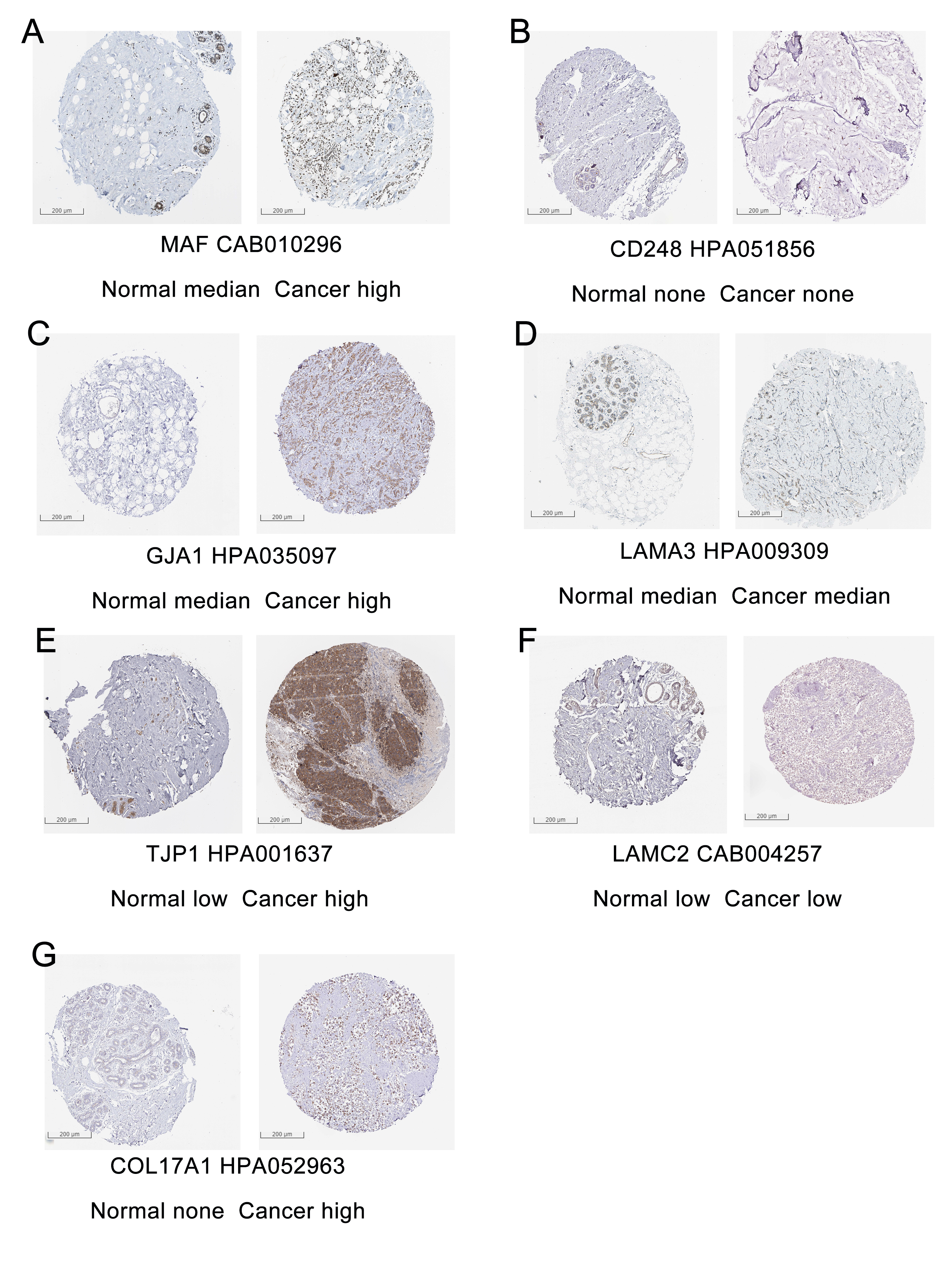

Supplement: Supplementary Figure 11 — The expression level of MAF (A), CD248 (B), GJA1 (C), LAMA3 (D), TJP1 (E), LAMC2 (F), and COL17A1 (G) in normal and BRCA tissue. [file Image_11.tif]

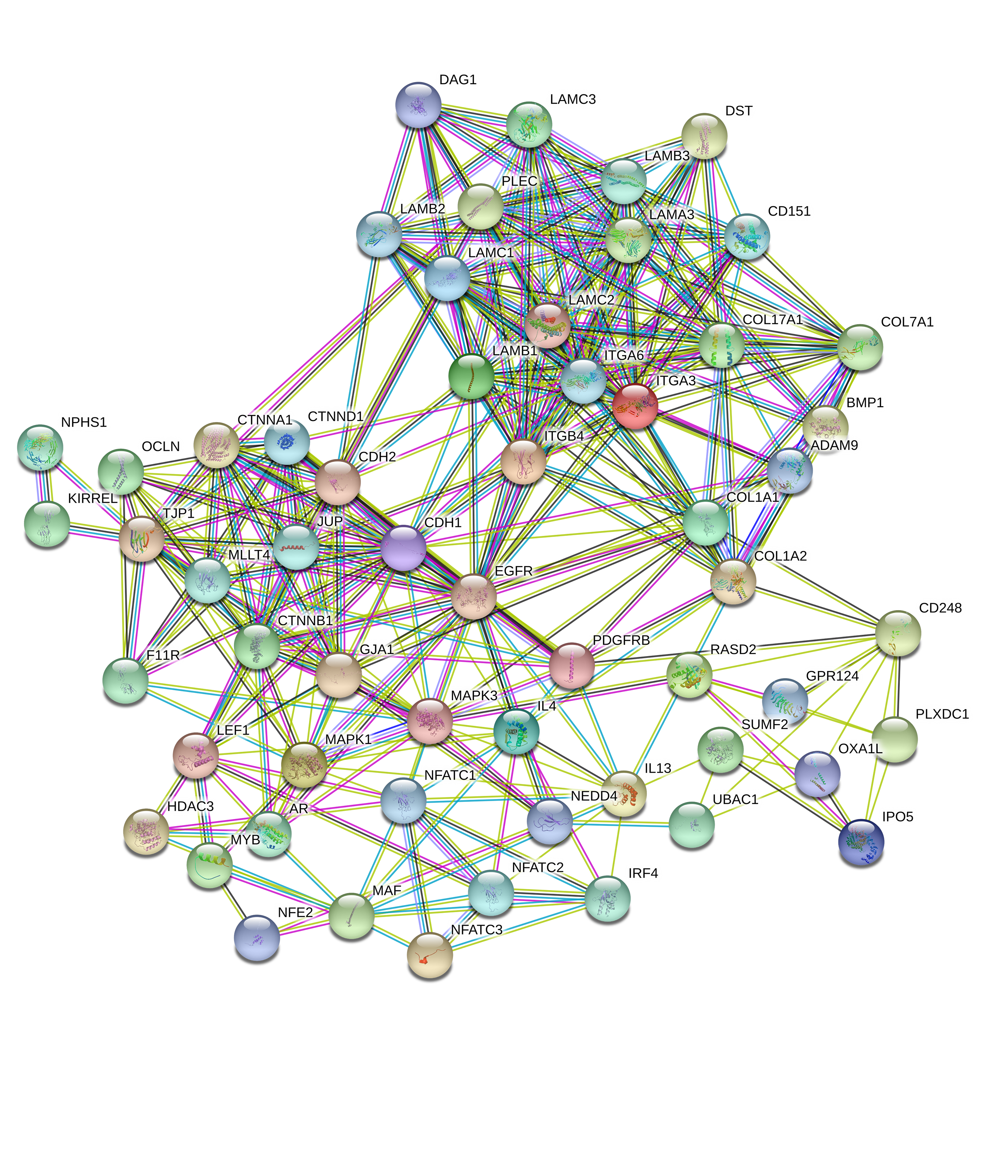

Supplement: Supplementary Figure 12 — The protein-protein interaction network of MAF, CD248, GJA1, LAMA3, TJP1, LAMC2, and COL17A1. [file Image_12.tif]
