# Supplementary material for: Construction of Bone Metastasis-Specific Regulation Network Based on Prognostic Stemness-Related Signatures in Breast Invasive Carcinoma
Source: Front Oncol. 2021 Jan 27;10:613333. doi: 10.3389/fonc.2020.613333 (PMC7875018; doi:10.3389/fonc.2020.613333)
Supplement: Supplementary file 14 [file Table_1.docx]

**Table S1.** Mutual Exclusivity of MAF, CD248, GJA1, LAMA3, TJP1, LAMC2 and COL17A1 in cBioportal database.

| **A** | **B** | **Neither** | **A Not B** | **B Not A** | **Both** | **Log2 Odds Ratio** | **p-Value** | **q-Value** | **Tendency** |
| --- | --- | --- | --- | --- | --- | --- | --- | --- | --- |
| GJA1 | LAMC2 | 3969 | 78 | 342 | 17 | 1.339 | 0.001 | 0.011 | Co-occurrence |
| GJA1 | COL17A1 | 4255 | 89 | 56 | 6 | 2.357 | 0.002 | 0.011 | Co-occurrence |
| LAMA3 | LAMC2 | 3966 | 81 | 342 | 17 | 1.283 | 0.002 | 0.011 | Co-occurrence |
| GJA1 | TJP1 | 4183 | 86 | 128 | 9 | 1.774 | 0.003 | 0.011 | Co-occurrence |
| TJP1 | COL17A1 | 4214 | 130 | 55 | 7 | 2.045 | 0.003 | 0.011 | Co-occurrence |
| TJP1 | LAMC2 | 3931 | 116 | 338 | 21 | 1.074 | 0.003 | 0.011 | Co-occurrence |
| LAMA3 | TJP1 | 4179 | 90 | 129 | 8 | 1.526 | 0.011 | 0.029 | Co-occurrence |
| CD248 | LAMA3 | 4178 | 130 | 90 | 8 | 1.514 | 0.011 | 0.029 | Co-occurrence |
| LAMC2 | COL17A1 | 3995 | 349 | 52 | 10 | 1.138 | 0.027 | 0.062 | Co-occurrence |
| MAF | GJA1 | 4230 | 81 | 90 | 5 | 1.537 | 0.037 | 0.078 | Co-occurrence |
| CD248 | GJA1 | 4179 | 132 | 89 | 6 | 1.094 | 0.076 | 0.145 | Co-occurrence |
| MAF | TJP1 | 4188 | 81 | 132 | 5 | 0.97 | 0.128 | 0.212 | Co-occurrence |
| MAF | CD248 | 4187 | 81 | 133 | 5 | 0.958 | 0.131 | 0.212 | Co-occurrence |
| LAMA3 | COL17A1 | 4249 | 95 | 59 | 3 | 1.185 | 0.159 | 0.238 | Co-occurrence |
| CD248 | TJP1 | 4133 | 136 | 135 | 2 | -1.151 | 0.189 | 0.265 | Mutual exclusivity |
| MAF | COL17A1 | 4260 | 84 | 60 | 2 | 0.757 | 0.342 | 0.438 | Co-occurrence |
| GJA1 | LAMA3 | 4216 | 92 | 95 | 3 | 0.533 | 0.354 | 0.438 | Co-occurrence |
| MAF | LAMC2 | 3969 | 78 | 351 | 8 | 0.214 | 0.402 | 0.469 | Co-occurrence |
| CD248 | LAMC2 | 3920 | 127 | 348 | 11 | -0.036 | 0.549 | 0.584 | Mutual exclusivity |
| MAF | LAMA3 | 4224 | 84 | 96 | 2 | 0.067 | 0.575 | 0.584 | Co-occurrence |
| CD248 | COL17A1 | 4208 | 136 | 60 | 2 | 0.045 | 0.584 | 0.584 | Co-occurrence |
