# Supplementary material for: Construction of Bone Metastasis-Specific Regulation Network Based on Prognostic Stemness-Related Signatures in Breast Invasive Carcinoma
Source: Front Oncol. 2021 Jan 27;10:613333. doi: 10.3389/fonc.2020.613333 (PMC7875018; doi:10.3389/fonc.2020.613333)
Supplement: Supplementary file 15 [file Table_2.docx]

**Table S2** Summary of multidimensional external validation results of gene expression base on multiple databases

|  | **MAF** | | **CD248** | | **GJA1** | | **LAMA3** | | **TJP1** | | **LAMC2** | | **COL17A1** | | **Results** |
| --- | --- | --- | --- | --- | --- | --- | --- | --- | --- | --- | --- | --- | --- | --- | --- |
|  | **N** | **B** | **N** | **B** | **N** | **B** | **N** | **B** | **N** | **B** | **N** | **B** | **N** | **B** |  |
| **GEPIA** | **↑** | **↓** | **↑** | **↑** | **↑** | **↑** | **↑** | **↑** | **↑** | **↑** | **↑** | **↑** | **↓** | **↑** | MAF, CD248, GJA1, LAMA3, TJP1 and LAMC2 highly expressed, while COL17A1 lowly expressed in normal. MAF lowly expressed, while CD248, GJA1, LAMA3, TJP1, LAMC2 and COL17A1 highly expressed in BRCA (figure S1). |
| **Oncomine** | **NA** | **↑** | **NA** | **-** | **NA** | **-** | **NA** | **↑** | **NA** | **-** | **NA** | **-** | **NA** | **↓** | MAF and LAMA3 highly expressed, while COL17A1 lowly expressed in BRCA (figure S2). |
| **PROGgeneV2** | **NA** | **↓** | **NA** | **↓** | **NA** | **-** | **NA** | **-** | **NA** | **↑** | **NA** | **↑** | **NA** | **↑** | TJP1, LAMC2 and COL17A1 highly expressed, while MAF and CD248 lowly expressed in BRCA (figure S3). |
| **UALCAN** | **↑** | **↓** | **↑** | **↓** | **↑** | **↑** | **↑** | **↓** | **↑** | **↑** | **↑** | **↓** | **↑** | **↓** | MAF, CD248, GJA1, LAMA3, TJP1, LAMC2 and COL17A1 highly expressed in normal. MAF, CD248, LAMA3, LAMC2 and COL17A1 lowly expressed, while GJA1 and TPJ1 highly expressed in BRCA (figure S4). |
| **Linkedomics** | **NA** | **-** | **NA** | **-** | **NA** | **-** | **NA** | **-** | **NA** | **-** | **NA** | **↓** | **NA** | **↓** | LAMC2 and COL17A1 lowly expressed in BRCA (figure S5). |
| **SurvExpress** | **↓** | **↑** | **↓** | **↑** | **↑** | **↓** | **-** | **-** | **-** | **-** | **↓** | **↑** | **↓** | **↑** | MAF, CD248, LAMC2 and COL17A1 lowly expressed, while GJA1 highly expressed in normal. MAF, CD248, LAMC2 and COL17A1 highly expressed, while GJA1 lowly expressed in normal (figure S6). |
| **cBioportal** | **NA** | **↑** | **NA** | **↑** | **NA** | **↑** | **NA** | **↑** | **NA** | **↑** | **NA** | **↑** | **NA** | **↑** | MAF, CD248, GJA1, LAMA3, TJP1, LAMC2 and COL17A1 highly expressed in BRCA (figure S7). |
| **GTEx** | **↓** | **NA** | **↑** | **NA** | **↑** | **NA** | **↓** | **NA** | **↓** | **NA** | **↓** | **NA** | **↓** | **NA** | MAF, LAMA3, TJP1, LAMC2 and COL17A1 lowly expressed, while CD248 and GJA1 highly expressed in normal (figure S8). |
| **UCSC xena** | **NA** | **↑** | **NA** | **↑** | **NA** | **↑** | **NA** | **↑** | **NA** | **↑** | **NA** | **↑** | **NA** | **↑** | MAF, CD248, GJA1, LAMA3, TJP1, LAMC2 and COL17A1 highly expressed in BRCA (figure S9). |
| **CCLE** | **NA** | **↓** | **NA** | **↓** | **NA** | **-** | **NA** | **↑** | **NA** | **↑** | **NA** | **↑** | **NA** | **↓** | MAF, CD248 and COL17A1 lowly expressed, while LAMA3, TJP1 and LAMC2 highly expressed in BRCA cell line (figure S10). |
| **Expression atlas** | **NA** | **↑** | **NA** | **↓** | **NA** | **↑** | **NA** | **↓** | **NA** | **↓** | **NA** | **↓** | **NA** | **↑** | MAF, GJA1 and COL17A1 highly expressed, while CD248, LAMA3, TJP1 and LAMC2 lowly expressed in BRCA. |
| **The human protein atlas** | **-** | **↑** | **ND** | **ND** | **-** | **↑** | **-** | **-** | **↓** | **↑** | **↓** | **↓** | **ND** | **↑** | MAF, GJA1, TJP1 and COL17A1 highly expressed, while LAMC2 lowly expressed in BRCA tissue. TJP1 and LAMC2 lowly expressed in normal breast tissue (figure S11). |

Note: “N” was defined as normal; “B” was defined as Breast invasive carcinoma;“↑” was defined as a significantly high-expressed gene; “↓” was defined as a significantly low-expressed gene; “NA” was defined as “Not available”; “ND” was defined as “Not detached”; “-” was defined as a gene with no significant difference in expression.

Abbreviations: BRCA, Breast invasive carcinoma; GTEx, Genotype-Tissue Expression; CCLE, Cancer Cell Line Encyclopedia; GEPIA, Gene Expression Profilling Interactive Analysis;
