# Supplementary material for: Construction of Bone Metastasis-Specific Regulation Network Based on Prognostic Stemness-Related Signatures in Breast Invasive Carcinoma
Source: Front Oncol. 2021 Jan 27;10:613333. doi: 10.3389/fonc.2020.613333 (PMC7875018; doi:10.3389/fonc.2020.613333)
Supplement: Supplementary file 16 [file Table_3.docx]

**Table S3** Summary of multidimensional external validation results of prognosis based on multiple databases

|  | **MAF** | **CD248** | **GJA1** | **LAMA3** | **TJP1** | **LAMC2** | **COL17A1** | **Results** |
| --- | --- | --- | --- | --- | --- | --- | --- | --- |
| **GEPIA** | OS *p* = 0.610  D/PF  *p* = 0.720 | OS *p* = 0.620  D/PF  *p* = 0.440 | NA | OS *p* = 0.091  D/PF  *p* = 0.530 | NA | NA | OS *p* = 0.014  D/PF  *p* = 0.069 | COL17A1 was significantly related to prognosis (figure S1). |
| **PROGgeneV2** | NKI M *p* = 0.472  GSE2990 M *p* = 0.125  GSE11121 M *p* = 0.013  GSE19783 OS *p* = 0.002  GSE3494 OS *p* = 0.044 | NA  GSE2990 M *p* = 0.036  GSE11121 M *p* = 0.135  GSE19783 OS *p* = 0.021  GSE3494 OS *p* = 0.017 | NKI M *p* = 0.005  GSE2990 M *p* = 0.007  GSE11121 M *p* = 0.003  GSE19783 OS *p* = 0.103  GSE3494 OS *p* = 0.001 | NKI M *p* = 0.008  GSE2990 M *p* = 0.102  GSE11121 M *p* = 0.231  GSE19783 OS *p* = 0.501  GSE3494 OS *p* = 0.054 | NKI M *p* = 0.095  GSE2990 M *p* = 0.057  GSE11121 M *p* = 0.060  GSE19783 OS *p* = 0.031  GSE3494 OS *p* = 0.010 | NKI M *p* = 0.058  GSE2990 M *p* = 0.018  GSE11121 M *p* = 0.610  GSE19783 OS *p* = 0.058  GSE3494 OS *p* = 0.953 | NKI M *p* = 0.178  GSE2990 M *p* = 0.086  GSE11121 M *p* = 0.682  GSE19783 OS *p* = 0.007  GSE3494 OS *p* = 0.838 | MAF, CD248, GJA1, LAMA3 and LAMC2 was significantly related to metastasis. MAF, CD248, GJA1, TJP1 and COL17A1 was significantly related to prognosis (figure S3). |
|  | NKI M *p* = 0.004  GSE2990 M *p* < 0.001  GSE11121 M *p* = 0.010  GSE19783 OS *p* = 0.019  GSE3494 OS *p* < 0.001 | | | | | | |  |
| **Linkedomics** | OS *p* = 0.971 | OS *p* = 0.834 | OS *p* = 0.269 | OS *p* = 0.066 | OS *p* = 0.249 | OS *p* = 0.038 | OS *p* = 0.001 | LAMC2 and COL17A1 were significantly related to prognosis (figure S5). |
| **SurvExpress** | Van OS *p* = 0.243  Van M *p* = 0.435  TCGA OS *p* = 0.048 | Van OS *p* = 0.561  Van M *p* = 0.444  TCGA OS *p* = 0.015 | Van OS *p* < 0.001  Van M *p* = 0.003  TCGA OS *p* = 0.622 | Van OS *p* = 0.047  Van M *p* = 0.006  TCGA OS *p* = 0.769 | Van OS *p* = 0.044  Van M *p* = 0.019  TCGA OS *p* = 0.021 | Van OS *p* = 0.974  Van M *p* = 0.103  TCGA OS *p* = 0.191 | Van OS *p* = 0.048  Van M *p* = 0.145  TCGA OS *p* = 0.304 | MAF, CD248, GJA1, LAMA3, TJP1 and COL17A1 were significantly related to overall survival, and GJA1, LAMA3 and TJP1 were significantly related to metastasis. Integrated genes were significantly related to prognosis and metastasis (figure S6). |
|  | Van OS *p* < 0.001  Van M *p* < 0.001  TCGA OS *p* < 0.001 | | | | | | |  |
| **cBioportal** | OS  *p* = 0.895  DF *p* = 0.376  PF *p* = 0.494 | OS *p* = 0.442  DF *p* = 0.757  PF *p* = 0.804 | OS *p* = 0.343  DF *p* = 0.154  PF *p* = 0.052 | OS *p* = 0.189  DF *p* = 0.071  PF *p* = 0.500 | OS *p* = 0.027  DF *p* = 0.941  PF *p* = 0.916 | OS *p* = 0.456  DF *p* = 0.110  PF *p* = 0.047 | OS *p* = 0.903  DF *p* = 0.407  PF *p* = 0.001 | TJP1 was significantly related to overall survival. LAMC2 and COL17A1 were significantly related to progression free (figure S7). |
|  | OS of integrated genes *p* = 0.503  DF *p* = 0.328  PF *p* = 0.271 | | | | | | |  |

Note: OS, Overall survival; DF, Disease free; PF, Progression free; M, metastasis; NA, not available;
